# Electron Capture Dissociation for Discovery Top-Down Proteomics of Peptides and Small Proteins on Chromatographic Time Scales

**DOI:** 10.1021/jasms.5c00116

**Published:** 2025-08-28

**Authors:** Lester S. Manly, Anne M. Roberts, Joseph S. Beckman, Blaine R. Roberts

**Affiliations:** † Department of Biochemistry, Emory University School of Medicine, Atlanta, Georgia 30322, United States; ‡ Molecular and Systems Pharmacology Graduate Program, Graduate Division of Biological and Biomedical Sciences, Laney Graduate School, 1371 Emory University, Atlanta, Georgia 30322, United States; § Linus Pauling Institute, 2694 Oregon State University, Corvallis, Oregon 97330, United States; ∥ Department of Neurology, Emory University School of Medicine, Atlanta, Georgia 30322, United States

## Abstract

Bottom-up proteomics introduces proteoform ambiguity due to the loss of connectivity between peptides and their original proteoforms. Top-down proteomics (TDP) removes the ambiguity through the direct identification and characterization of intact proteoforms and their respective post-translational modifications (PTM). Electron capture dissociation (ECD) is an efficient and gentle peptide and protein fragmentation strategy that can be used for both bottom-up and top-down approaches. Here, we used an Agilent 6550 Q-TOF mass spectrometer retrofitted with an e-MSion ECD cell. Top-down sequencing capabilities of the cell were evaluated by sequencing of intact peptides and proteins on high-performance liquid chromatography (HPLC) time scales. Amyloid beta 1-40 (Aβ40) was first tested due to its pathophysiological role in Alzheimer’s disease and served as our large peptide standard. We sequenced Aβ40 via reverse-phase HPLC-MS and achieved 95% sequence coverage on chromatographic time scales utilizing a data-dependent acquisition (DDA)-based method. Acetone-precipitated protein extracts from human brain were then separated by HPLC and analyzed with a DDA method, which identified 16 proteoforms between 2 and 17 kDa with sequence coverage ranging from 7 to 90% based on proteoform size and composition. In addition to proteoform identification, ECD fragmentation distinguished multiple isoaspartate modifications from aspartate, as well as accurately differentiating leucine from isoleucine residues directly from the human brain sample. Here, we observed isoaspartate within a thymosin beta-4 proteoform. Additionally, we demonstrated the differentiation of leucine and isoleucine within a subunit of ubiquitin. This study advances the application of LC-Q-TOF instrumentation for discovery-based top-down proteomics utilizing ECD as enabled by the e-MSion ECD cell.

## Graphical abstract

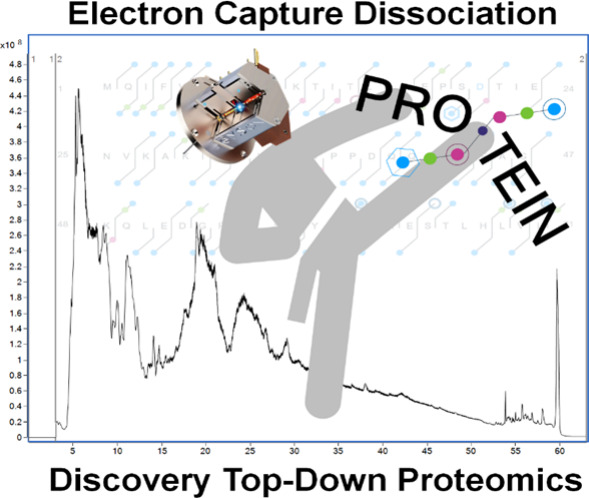

## Introduction

A detailed molecular understanding of the structure and function of proteins is one of the ultimate goals of proteomics. Achieving this requires the measurement of several properties of a protein e.g., abundance levels, post-translational modifications (PTMs), noncovalent cofactors, localization and protein–protein interactions. And then, ideally, doing this on a global proteome scale. However, significant development and innovation within the field are still required for a comprehensive solution.

Bottom-up proteomics (BUP) has made progress toward addressing these questions, achieving near complete coverage of a given proteome, yielding measurement of protein abundance and a limited insight into PTMs and other proteome inferences.^1–4^ However, a key limitation of the BUP approach – which requires the digestion of proteins into peptides – is the inability to fully reconstruct the original proteoforms present. Although BUP can identify many peptides with PTMs from a protein, the approach does not provide direct information about the combination of the PTMs in the original intact proteoform. For example, if there are multiple sites of phosphorylation of a given protein does a proteoform exist with all the phosphorylation or is there a mixture of proteoforms with various combinations of PTM? Understanding the distribution of multiple PTMs on a given protein may add an additional layer of complexity to how modifications regulate function and localization. A barcode rather than a single on or off signal. The ambiguity introduced via proteolytic digestion prevents reassembly of the peptide parts into the proteoforms that were in the sample and top-down proteomics (TDP) begins to address this challenge.

TDP aims to bridge this gap by directly measuring proteoforms. However, the technology available for TDP is not as widely available or established as with current BUP approaches. One of most common fragmentation technologies used in proteomics is collision-induced dissociation (CID; also known as collisionally activated dissociation, CAD).^5^ CID fragments peptide and protein ions by accelerating them into a cell filled with neutral gas molecules, where they undergo inelastic collisions. These collisions convert a portion of the ions’ kinetic energy into internal vibrational energy, which is redistributed throughout the molecule in an ergodic manner, i.e., slow heating of the molecule. This ergodic vibrational excitation promotes cleavage along the peptide backbone, resulting primarily in the formation of *b*- and *y*-type fragmentions.^6,7^ However, CID has inherent limitations, including difficulty in preserving labile PTMs and limited applicability for intact proteins due to the majority of the fragmentation occurring at either the N- or C- termini.^8,9^ A common complementary fragmentation method to CID is electron transfer dissociation (ETD).^10^ ETD is a gas-phase chemical reaction in which an electron is transferred from radical anions to protonated cations.^11^ The resulting radical cation is unstable, leading to fragmentation of the peptide backbone and yielding *c*- and *z*- fragment ions. One major advantage of ETD is its ability to preserve labile PTMs, such as phosphorylation, glycosylation, acetylation, and nitration.^12^ Additionally, ETD can fragment intact proteins, enabling top-down sequencing. One challenge with ETD is the phenomenon of electron transfer to the protein but the molecule fails to produce fragments, referred to as electron transfer with no dissociation (ETnoD).^13,14^ The second, is the requirement of ETD to have relatively long reaction times to allow transfer from the anion reagent this limits its application to mass spectrometers with ion-trapping capabilities.

Related to ETD is electron capture dissociation (ECD). Like ETD, ECD fragments peptide and protein ions through the respective cations capturing a free electron, directly produced by the instrument, which cause the cation to become an unstable radical resulting in subsequent peptide backbone fragmentation and yielding *c*- and *z*- fragment ions. The development of the e-MSion ECD cell by Voinov and Barofsky^15^ provides an alternative fragmentation method for both linear mass spectrometers, such as quadrupole time-of-flight (Q-TOF)^16^ and triple quadrupoles (QqQ),^17^ as well as for ion trapping instruments, including quadrupole orbitrap instruments.^18,19^ Before the development of ECD cells compatible with Q-TOF instruments, ECD was limited to FT-ICR platforms. One key reason for the success of the e-MSion ECD cell is that low-energy electrons are confined directly within the molecular ion beam path using magnetic and electrostatic fields. An advantage of ECD over ETD is that ECD reaction occurs on the microsecond (μs) time scale, whereas ETD typically takes milliseconds (ms), eliminating the need for an ion-trapping mechanism for efficient fragmentation (Figure 1A). The e-MSion ECD cell, the produced electrons are introduced colinearly with the ion beam, i.e., axial electron injection, and ECD fragmentation of peptides or proteins occurs as the analytes fly through the cell, which is approximately 5 μs. A key limitation of ETD is that the required reaction time is on the order of 1–100 ms. Additionally, the ECD fragmentation mechanism produces a broad range of fragments, including the typical *c*- and *z*- ions observed in ETD, as well as secondary *w*- and *d*- side chain fragment ions, which provide valuable information for assigning side-chain structures, such as distinguishing between isoleucine and leucine.^16,20^

**Figure 1:**
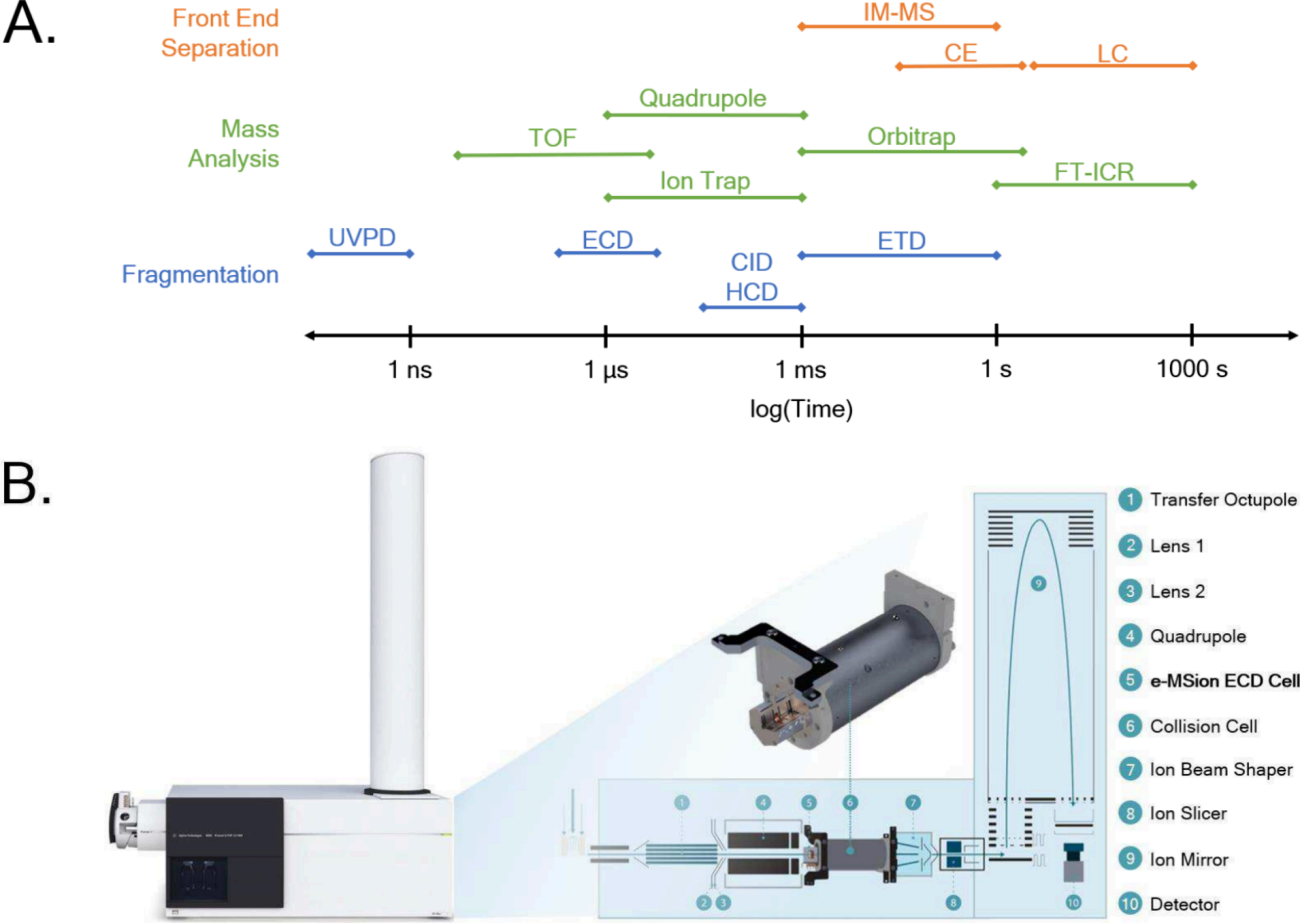
Comparison of relative operational time scales (A) between commonplace front-end separation, mass analysis, and fragmentation modalities. Diagram of an Agilent 6550 Q-TOF mass spectrometer modified with an e-MSion electron capture dissociation cell (B), which has been added to the quadrupole side of a shortened standard collision cell found in Agilent Q-TOFs. IM-MS = Ion Mobility Mass Spectrometry; CE = Capillary Electrophoresis; LC = Liquid Chromatography; TOF = Time Of Flight; FT-ICR = Fourier-Transform Ion Cyclotron Resonance; UVPD = Ultraviolet Photodissociation; ECD = Electron Capture Dissociation; CID = Collision Induced Dissociation; HCD = Higher-Energy Collisional Dissociation; ETD = Electron Transfer Dissociation.

ECD can also be described by the amount of kinetic energy of the free electron, also known as electron energy (eV), prior to capture for analyte dissociation or charge state reduction.^21^

This energy is typically controlled by adjusting the potential difference in the reaction cell. Low-energy ECD uses electrons at ∼0.1–0.3 eV, while hot ECD involves electrons at ∼1–10 eV. Electron-induced dissociation (EID) in positive mode uses higher energies of ∼10–20 eV, and in negative mode, similar energy levels (∼15–20 eV) lead to electron detachment dissociation (EDD). Higher electron energies generally result in more extensive fragmentation and greater sequence coverage, but with increased risk of losing labile post-translational modifications (PTMs). Low-energy ECD is the most common application for the e-MSion ECD cell, which offers efficient fragmentation with potential for high sequence coverage and confident PTM localization for peptides and proteins. As mentioned prior, the e-MSion ECD cell has the capability of providing information to distinguish isobaric residues, such as isoleucine and leucine and aspartate and aspartate, within peptides and proteins using low-energy ECD.^16^ In this study we only explored the use of low-energy electrons.

Here, we describe the retrofitting of an Agilent 6550 Q-TOF mass spectrometer with an e-MSion ECD cell (Figure 1B). The goal of this study was to investigate if ECD, as the sole fragmentation method, was sufficient protein sequencing on chromatographic time scales. We demonstrate that Q-ECD-TOF can operate on liquid chromatographic time scales in an automated manner using a data-dependent acquisition strategy analogous to those employed in BUP. The e-MSion ECD cell enabled 95% sequence coverage of full-length synthetic amyloid beta 1-40 and proteoform identification of several intact proteins extracted from human brain tissue across the chromatographic gradient utilizing an ECD as the sole fragmentation method.

## Experimental Section

### Agilent 6550 Q-TOF and e-MSion ECD Cell Design and Configuration

The prototype e-MSion ECD cell was installed in an Agilent 6550 Q-TOF mass spectrometer, as shown in Figure 1B, by replacing the standard collision cell with the modified collision cell with a rear facing ECD cell, which will referred to as the “e-MSion ECD cell” onward (e-MSion, Inc.). After the hardware installation and re-establishment of the vacuum, the e-MSion ECD cell was activated using default voltage, which allowed transmission of ions to the TOF analyzer. The Q-TOF was then tuned and calibrated using the Agilent low-concentration tune mix (ESI-L) at 3,200 *m*/*z*, in 2 GHz high-sensitivity mode, and the automated tuning software (MassHunter V.08.00.SP2, Agilent Technologies, Inc.). Ion transmission and ECD fragmentation efficiency were optimized by monitoring the signal from the peptide Substance P (Sigma-Aldrich). First, total transmission of Substance P was optimized using the ramp function on the collision cell entrance, followed by manual adjustment of the voltages on lenses 3–6. The ECD efficiency was optimized by isolating the 2+ precursor of Substance P at 647.4 *m*/*z* and monitoring the intensity of the 624 *m/z c*_5_^1+^ fragment ion. Total ECD efficiency was determined by summing the areas of all *c-/z-* fragment ions of substance P and dividing by the total precursor ion intensity. Typically, efficiency ranges between 1.0% and 3.0% for this cell. We used a transmission-based MS1 profile by placing the filament on standby and an MS2 profile that was tuned for ECD fragmentation using Substance P with an ECD fragmentation >0.2%. This MS2 profile is efficient for peptide and small protein fragmentation.

### LC-MS/MS for Intact Top-Down Proteomics

The HPLC system used was an Infinity II UHPLC, equipped with an autosampler featuring Peltier cooling maintained at 4 °C, binary pumps, and a thermostatically controlled column compartment. Proteins were resolved using a Poroshell Extend 300 C18 column (2.1 × 75 mm, Agilent Technologies, Inc.). To maximize recovery and limit protein adsorption, the column was maintained at 8 °C. The solvents utilized to form gradients were 0.1% formic acid (Sigma-Aldrich; LC-MS grade) in doubly distilled water (buffer A) and 0.1% formic acid in acetonitrile (Sigma-Aldrich; LC-MS grade). Two gradients were used for the separation of intact proteins. To sequence amyloid beta 1-40, a 10 min method at a flow rate of 0.4 mL/min (Time (min), %B): 0, 2.5; 1, 25; 5, 80; 7, 80; 7.5, 2.5; 10, 2.5 was utilized. To resolve the semicomplex brain extract, the sample was chromatographically separated using a 60 min gradient (Time (min), %B): 0, 3; 3, 3; 5, 20; 50, 60; 55, 90; 58, 90; 59, 3. The 6550 QTOF instrument acquisition parameters used for both data-dependent TDP experiments are listed in Supplemental Figure 1 for the amyloid beta 1-40 sequencing experiment and Supplemental Figure 2 for proteoform discovery in the semicomplex brain extract experiment. The primary data-dependent acquisition (DDA) parameters that were adjusted for both experiments were “Max Precursor Per Cycle” which was set to 2, “Precursor Threshold – Abs. Threshold” which was set to 25,000 counts for the amyloid beta 1-40 experiment then later increased to 75,000 human brain extract experiment, and “Precursor Charge-State Selection and Preference” where we enabled >3 and Unk charge states.

### Synthetic Full Length Amyloid Beta 1-40

The amyloid beta 1-40 used was synthesized and purified in-house as previously published.^22^

### Preparation of Human Brain Extract

All experiments were conducted with approval from the University of Melbourne Human Ethics Committee (ID 1750801). Brain tissue was obtained from the Victorian Brain Bank and included samples from individuals with mixed neurological diseases, including Alzheimer’s and Parkinson’s, as well as control tissue.

Fresh-frozen brain tissues were initially stored at −80̊C but warmed to approximately 6̊C due to a power failure that caused the freezer to turn off, allowing the tissue to gradually warm over 2 days. The tissue was then transferred to a wet ice bath for 1–2 h before homogenization. Homogenization of 4.6 kg of brain tissue, sourced from 10–15 individuals, was performed in batches of 300–500 g using a 2 L stainless steel blender (Waring Commercial; Torrington, CT). Tissue was mixed at a 1:2 ratio with Tris-buffered saline (TBS; 100 mM Tris, pH 8.0, 100 mM NaCl), such that 300 g of tissue was blended with 600 mL of TBS. The mixture was blended at low speed for 30 s, followed by 1 min on high speed. The homogenate was clarified by centrifugation in 1 L centrifuge tubes at 10,000 × g for 20 min at 4̊C. The resulting TBS supernatant was combined with chilled (−20̊C) acetone at a final 4:1 ratio in 20 L high-density polyethylene containers and incubated overnight at 4̊C. The following day, the acetone solution was centrifuged, decanted, and the resulting pellet was resuspended in 200 mL of double distilled water by incubation on ice with gentle stirring for 2 h. The solution was then centrifuged at 20,000 × g for 30 min, and the resulting supernatant was stored at −20̊C until LC-MS/MS analysis.

Protein concentrations were determined using a micro BCA assay (Thermo Fisher Scientific). Thirty μg of protein was injected for analysis.

### Data Analysis

Chromatographic and spectral data were analyzed directly using MassHunter Qualitative Analysis 10.0 (Agilent Technologies, Inc.). Targeted sequencing analysis was conducted using ExDViewer version 4.6.28 (Agilent Technologies, Inc.). For the amyloid beta 1-40 sequencing experiment, MS1 and MS2 mass error tolerances were set to 10 ppm. All fragment ion matches were subsequently reviewed manually inspected in ExDViewer to ensure accuracy. Criteria for validation included the presence of multiple isotopologues per match, evaluation of the averagine fit quality for each matched isotopic distribution, and confirmation that the averaged MS2 mass error across isotopic peaks was less than 10 ppm. For the human brain pro extract experiment, proteoform identification was performed using the TopPIC suite version 1.7. Feature detection was conducted with TopFD version 1.7.8, where the fragmentation method was set to “ETD” to include c- and z-ion fragments. TopPIC version 1.7.8 was used for the top-down proteoform search, utilizing the *Homo sapiens* canonical and reviewed FASTA file from UniProt (retrieved in February 2025). The search mass error tolerance was set to 10 ppm, the fragmentation method was adjusted to “ETD”, and the false discovery rate (FDR) cutoffs for both spectrum and proteoform levels were set to 0.01. Additionally, N-terminal modifications, including methionine loss and/or acetylation, were included in the search parameters. Sequence coverage for the identified proteoforms was determined using ExDViewer for targeted sequencing analysis using mass tolerances of 5 ppm for MS1 and 10 ppm for MS2.

## Results and Discussion

### Top-Down Sequencing of Amyloid Beta 1-40 Using ECD on LC Time Scales

We aimed to start beyond the typical mass range of tryptic peptides characteristic of BUP, which does not extend past 4 kDa.^23^ Specifically, we began with Amyloid Beta 1-40 (Aβ40, 4.3 kDa), a key proteoform involved in the pathophysiology of Alzheimer’s Disease (AD). To simulate a real-world application, all proteins were sequenced directly as they eluted from the LC column, rather than by direct infusion, which is common approach in TDP studies. Precursor charge states were selected using data-dependent acquisition (DDA), targeting the top two most abundant ions. ECD MS/MS spectra were acquired with a one-second accumulation time per scan, resulting in a total cycle time of 2.225 s. This setup was designed to evaluate the efficiency of the e-MSion ECD cell for sequencing small proteins on chromatographic time scales. For Aβ40, we focused on the most abundant charge state observed under our instrument conditions: 6+ (*m*/*z* 722).

Extracted ion chromatogram of the 6+ charge revealed that Aβ40 had peak characteristics of full width at half-maximum of 0.091 min and peak width of 0.223 min (Figure 2A). This elution time scale provided the instrument approximately 13 s for isolation and fragmentation, which mirrors what occurs in real-world experiments.

**Figure 2:**
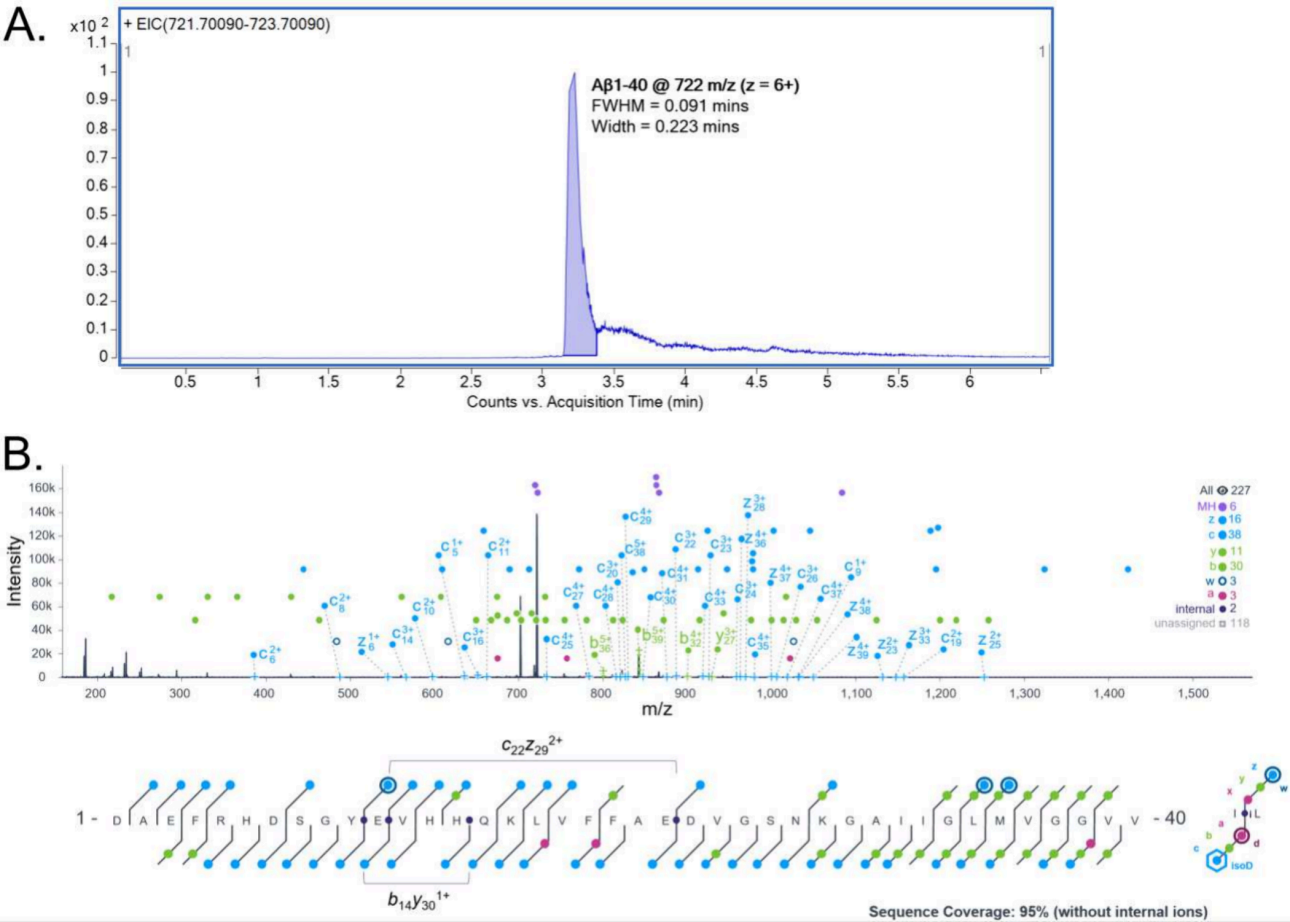

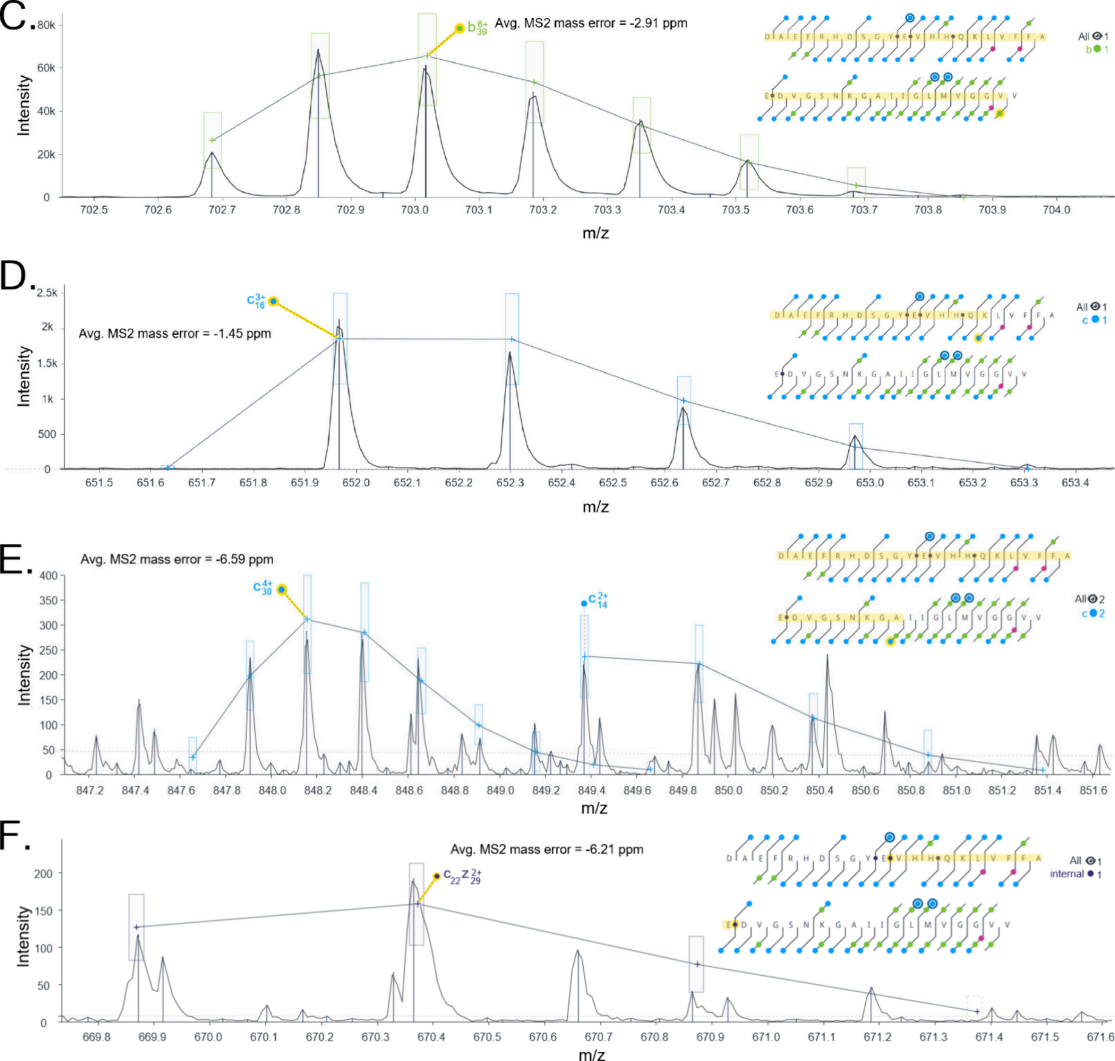
Sequencing of synthetic amyloid beta 1-40 (Aβ40) on a chromatographic time scale. Extracted ion chromatogram (A) of the 6+ charge state of Aβ40 for ECD MS/MS. MS/MS spectra and sequence coverage map with annotation of fragments contributing to sequence coverage (B) with all fragment ions considered and internal ion annotation using ExDViewer. Brackets on sequence coverage map indicate internal ion(s). Zoomed in annotation of the most abundant fragment *b*_39_^6+^ (C), most abundant ECD fragment *c*_16_^3+^, highest annotated MS2 error fragment *c*_30_^4+^ and overlap with *c*_14_^2+^ (E), and example of an internal ion annotation *c*_22_ *z*_29_^2+^ (F). The zoomed in annotations all have insets that indicate the location of the fragment or internal ion with the Aβ40 sequence. The floating-colored boxes indicate isotopes that match the respective fragment averagine and empty dotted boxes indicate no match. Red dotted line indicates signal-to-noise cutoff.

Averaging just three scans of the eluting chromatographic peak yielded 95% sequencing coverage when all fragment types were considered (Figure 2B). This high level of coverage demonstrates the quality of fragmentation achievable through ECD with minimal sampling. This sequencing performance compares favorably to other LC-MS/MS systems, where a significant portion of the field utilize high-resolution orbitrap instruments.^24–26^ To our knowledge, this work represents one of the highest degrees of sequence coverage of full-length Aβ achieved using an LC-MS/MS system. To assess annotation confidence, we used ExDViewer to examine key fragment ions, including the most abundant overall fragment *b*_39_^6+^ (Figure 2C) and the most intense ECD fragment *c*_16_^3+^ (Figure 2D), demonstrating ExDViewer’s utility for targeted MS/MS data analysis. A summary of all fragment annotations contributing to Aβ40 sequence coverage is provided in Table 1, with all associated MS2 mass errors below 10 ppm. The fragment ion with the highest observed mass error, *c*_30_^4+^, showed a deviation of −6.59 ppm (Figure 2E). We also highlighted an internal ion annotation by zooming into the *c*_22_ *z*_29_^2+^ internal fragment observed in this data set.

**Table 1:**
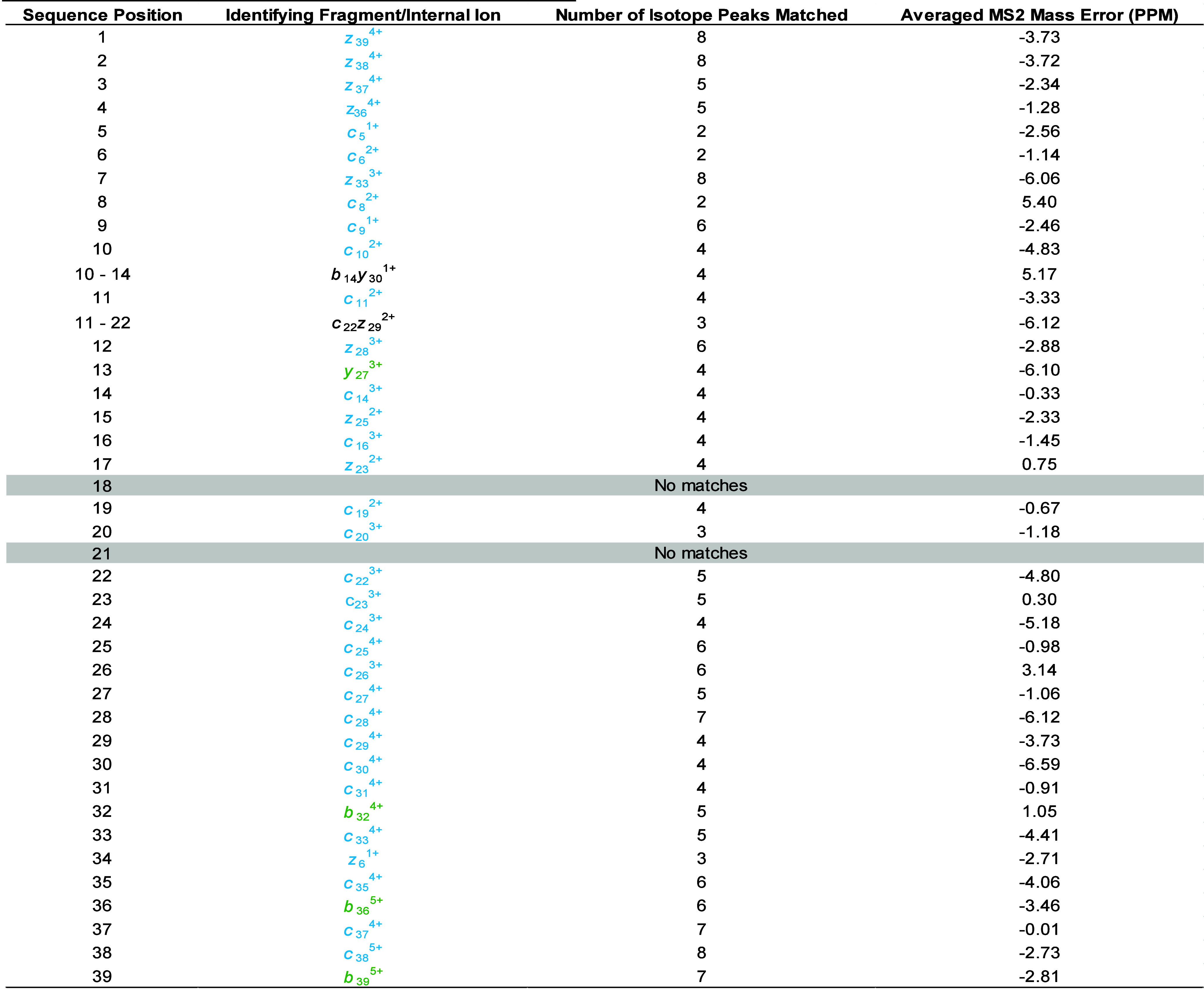
Fragment and Internal Ion Annotations Contributing to the Sequence Coverage of Synthetic Amyloid Beta 1-40 (Aβ40) as Assigned by ExDViewer and after Manual Inspection of Accuracy of Annotations

Future directions will include using our LC-Q-ECD-TOF system for the top-down characterization of endogenous Aβ to identify and localize PTMs leveraging the sequencing capabilities of ECD. Here, we demonstrated the Agilent 6550 equipped with ECD was able to isolate and sequence our intact Aβ40 as the sample was introduced by LC. With this information, we proceeded to evaluate the potential of ECD in discovery TDP using a sample containing multiple proteins.

### Discovery Top-Down Sequencing of an Intact Protein Sample from Human Brain Using ECD on LC Time Scales

To test the applicability of top-down sequencing multiple proteins along a chromatographic gradient, we analyzed proteins extracted from human brain tissue using acetone precipitation (Supplemental Figure 3). Acetone-precipitated proteins were resuspended in double distilled water and 30 μg, in 20 μL, was injected onto the column. The instrument was set up to automatically select two targets to isolate and collect 1 s ECD MS/MS spectra per precursor. To limit the amount of time the instrument spent collecting 1+ charge peptides and other chemical species, we limited the mass range (200–3,200 *m*/*z*) and had the system ignore the 1+ charge state. In addition, we increased the minimum intensity threshold to 75,000 counts to increase the abundance of the resulting product ions (Supplemental Figure 2). After a database search of the collected data using TopPIC suite, we identified 16 unique and high-confidence proteoforms (Table 2) over the course of the 60 min gradient (Figure 3). The proteoforms we identified ranged from 2 and 17 kDa with an overall sequence coverage across the mass range up 90% and coverage of N- and C- terminal sequence tags, all acquired on HPLC time scales. The compact range of the proteoforms was anticipated due to the acetone precipitation and resuspension back into water, all of which would favor peptides and smaller proteins. The lowest molecular mass of a proteoform we identified was thymosin beta-4 [25–40] fragment at 1.9 kDa at a charge state of 3+ (Figure 3B), with the largest being calmodulin-1 [2–149] at 16.7 kDa at a charge state of 15+ (Figure 3D). Here, we highlighted an example of fragment ion annotation for identification of both thymosin beta-4 (Figure 3C) and calmodulin-1 (Figure 3E), which would be the highest intensity fragment ion contributing to the sequence tag match of the respective proteoform. Interestingly, we did not match to the precursor for calmodulin-1 as TopPIC identified as 43.0721 Da between the residues 126–139: M.[Acetyl]­ADQL­TEEQIA­EFKEA­FSLFDKD­GDGTITTK­ELGTVM­RSLGQNP­TEAELQD­MINEVD­ADGNGT­IDFPEFLT­MMARKM­KDTDSEE­EIREAFR­VFDKD­GNGY­ISAAEL­RHVM­TNLGEK­LTDEEV­DEMI­(READ­IDGDGQ­VNYE)­[+43.0721]­EFVQM­MTAK. Unfortunately, were not able to get a sequence match via variable PTM search with ExDViewer with consideration of the following PTMs: Carboxylation (Unimod Accession: 299 – DE), Ethaolamine (Unimod Accession: 735 – DE), nor Carbamylation (Unimod Accession: 5 – RY). Overall, we observed N-terminal methionine loss and/or acetylation common in eukaryotic samples. We did not observe the identification of phosphorylation (STY), SUMOylation (K), nor nitration (WY) modified proteoforms, suggesting enrichment would be required to directly measure these proteoforms.

**Figure 3:**
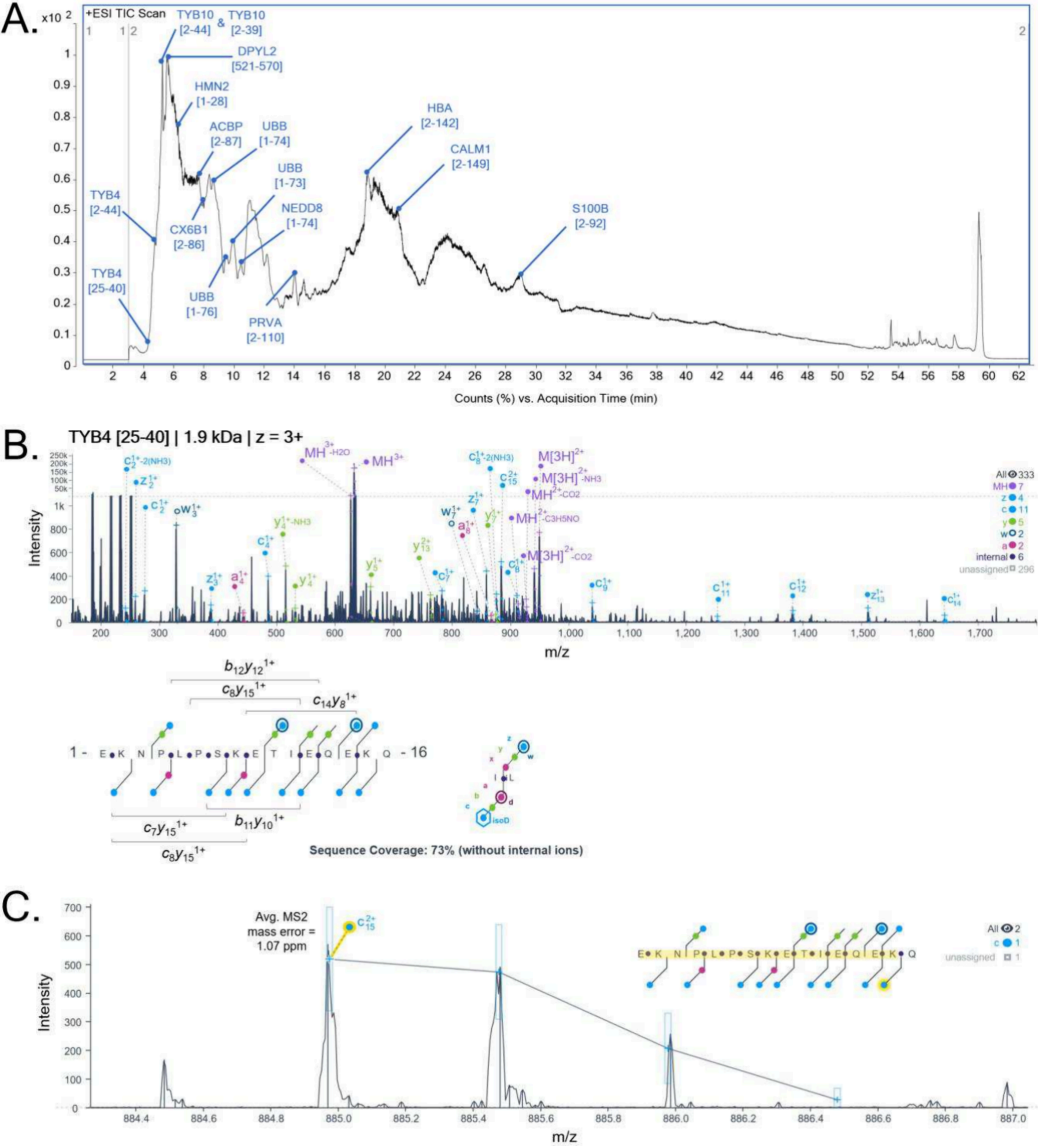

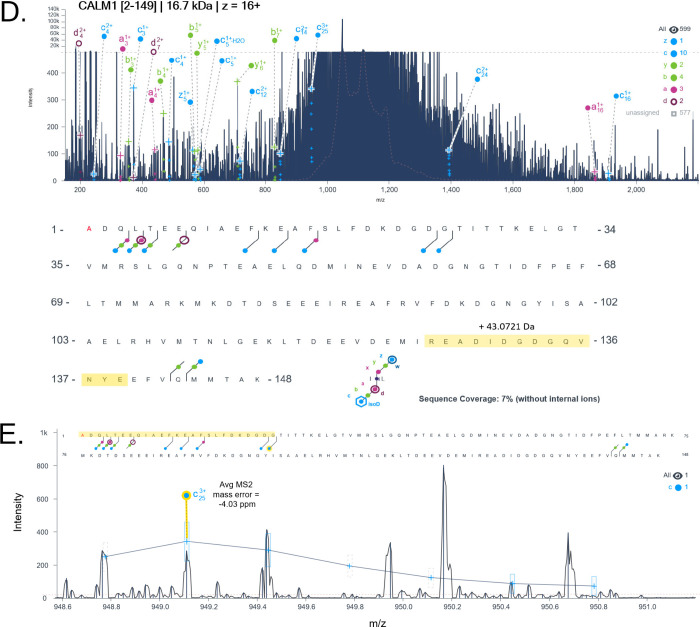
Annotated chromatogram (A) of acetone-precipitated intact protein sample from human brain. The numbers in parentheses indicate sequence location. MS/MS spectra and sequence coverage map of the lightest identified proteoform thymosin beta-4 [25–40] at 1.9 kDa (B) to the heaviest identified proteoform of calmodulin-1 [2–149] at 16.7 kDa (D). Examples of annotations for the a most abundant fragment for thymosin beta-4 [25–40] (C) and calmodulin-1 [2–149] (E) are provided. Insets indicate length of fragment. Red-colored letters in sequence coverage map indicate N-terminal acetylation. Charge state included in panel indicates what was identified by TopPIC untargeted search and used for targeted search with ExDViewer.

**Table 2:** TopPIC Identified Proteoforms Present within an Intact Protein Sample of Acetone-Precipitated from Human Brain Sequenced with ECD Using an Overall Mass Error of 10 ppm with FDR of 0.01^a^

Protein	Protein Description	Protein Accession	Sequence Position	Sequence Coverage (%)	Observed Mass (Da)	Retention Time (min)
sp|P62328|TYB4_HUMAN	Thymosin beta-4 OS = *Homo sapiens* OX = 9606 GN = TMSB4X PE = 1 SV = 2	P62328	25-40	73	1896.98	4.49
			2-44	90	4960.49	5.32
sp|P63313|TYB10_HUMAN	Thymosin beta-10 OS = *Homo sapiens* OX = 9606 GN = TMSB10 PE = 1SV = 2	P63313	2-44	31	4734.41	5.39
			2-39	27	4361.23	5.42
sp|Q16555|DPYL2_HUMAN	Dihydropyrimidinase-related protein 2 OS = *Homo sapiens* OX = 9606 GN = DPYSL2 PE = 1 SV = 1	Q16555	521-570	45	5304.79	5.63
sp|P0CJ69|HMN2_HUMAN	Humanin-like 2 OS = *Homo sapiens* OX = 9606 GN = MTRNR2L2 PE = 2 SV = 1	P0CJ69	1-28	7	3022.63	6.59
sp|P07108|ACBP_HUMAN	Acyl-CoA-binding protein OS = *Homo sapiens* OX = 9606 GN = DBI PE = 1 SV = 2	P07108	2-87	35	9948.99	7.82
sp|P14854|CX6B1_HUMAN	Cytochrome c oxidase subunit 6B1 OS = *Homo sapiens* OX = 9606 GN = COX6B1 PE = 1 SV = 2	P14854	2-86	19	10093.77	8.04
sp|P0CG47|UBB_HUMAN	Polyubiquitin-B OS = *Homo sapiens* X = 9606 GN = UBB PE = 1 SV = 1	P0CG47	1-74	70	8445.57	9.00
			1-76	17	8559.62	9.59
			1-73	75	8289.47	10.08
sp|Q15843|NEDD8_HUMAN	Ubiquitin-like protein NEDD8 OS = *Homo sapiens* OX = 9606 GN = NEDD8 PE = 1 SV = 1	Q15843	1-73	10	8284.52	10.55
sp|P20472|PRVA_HUMAN	Parvalbumin alpha OS = *Homo sapiens* OX = 9606 GN = PVALB PE = 1 SV = 2	P20472	2-110	21	11961.98	14.17
sp|P69905|HBA_HUMAN	Hemoglobin subunit alpha OS = *Homo sapiens* OX = 9606 GN = HBA1 PE = 1 SV = 2	P68871	2-142	14	15421.02	19.01
sp|P0DP23|CALM1_HUMAN	Calmodulin-1 OS = *Homo sapiens* OX = 9606 GN = CALM1 PE = 1 SV = 1	P0DP23	2-149	7	16780.88	21.74
sp|P04271|S100B_HUMAN	Protein S100-B OS = *Homo sapiens* OX = 9606 GN = S100B PE = 1 SV = 2	P04271	2-92	36	10617.02	29.14

a The sequence coverage percentage was determined using ExDViewer using the averaged mass errors of 5 ppm for MS1 and 10 ppm for MS2.

One benefit of utilizing an electron associated dissociation method, either ECD or ETD, is the ability of the fragmentation method to identify aspartate residues modified through isomerization to isoaspartate through fragmentation.^27,28^ Aspartate and isoaspartate (isoD) can be identified by detecting O’Connor fragments,^29^ where fragmentation of isoD residues results in a shift of 57 Da for the respective c- (+57 Da) or z- (−57 Da) ions.^30^ IsoD formation has been associated with reduced structural stability and functionality of proteins leading to subsequent degradation^31^ or potentially being enzymatically repaired with protein L-isoaspartyl methyltransferase (PIMT),^32^ which can also be used to label isoD.^33^ Relating back to Aβ and AD, isoD has been identified in Aβ with isoD at positions 1, 7, and 23 were deposited in senile plaques and amyloid-bearing vessels and prior work from our lab indicates up to 80% of Aβ1–15 was isomerized at positions 1 or 7 detected using ion mobility mass spectrometry.^34^ Here we were able to identify a thymosin beta-4 proteoform that contains an isoD residue at position 5 (Figure 4A). Within this proteoform, we provided an example comparison of side group fragmentation identification, *w*- ion, of aspartate (Figure 4B) and *c*- ion +57 Da mass shift identification of isoD (Figure 4C). The mass shift of −45 Da with aspartate fragmentation via ECD is the result of a radical carboxyl group loss. Thymosin beta-4 is an endogenous peptide implicated in inhibiting inflammation and apoptosis while increasing angiogenesis^35^ and it has been found to prevent amyloid deposition in an AD mouse model.^36^ However, the specific effects of the presence of isoD residues on thymosin beta 4 residues have yet to be explored.

**Figure 4:**
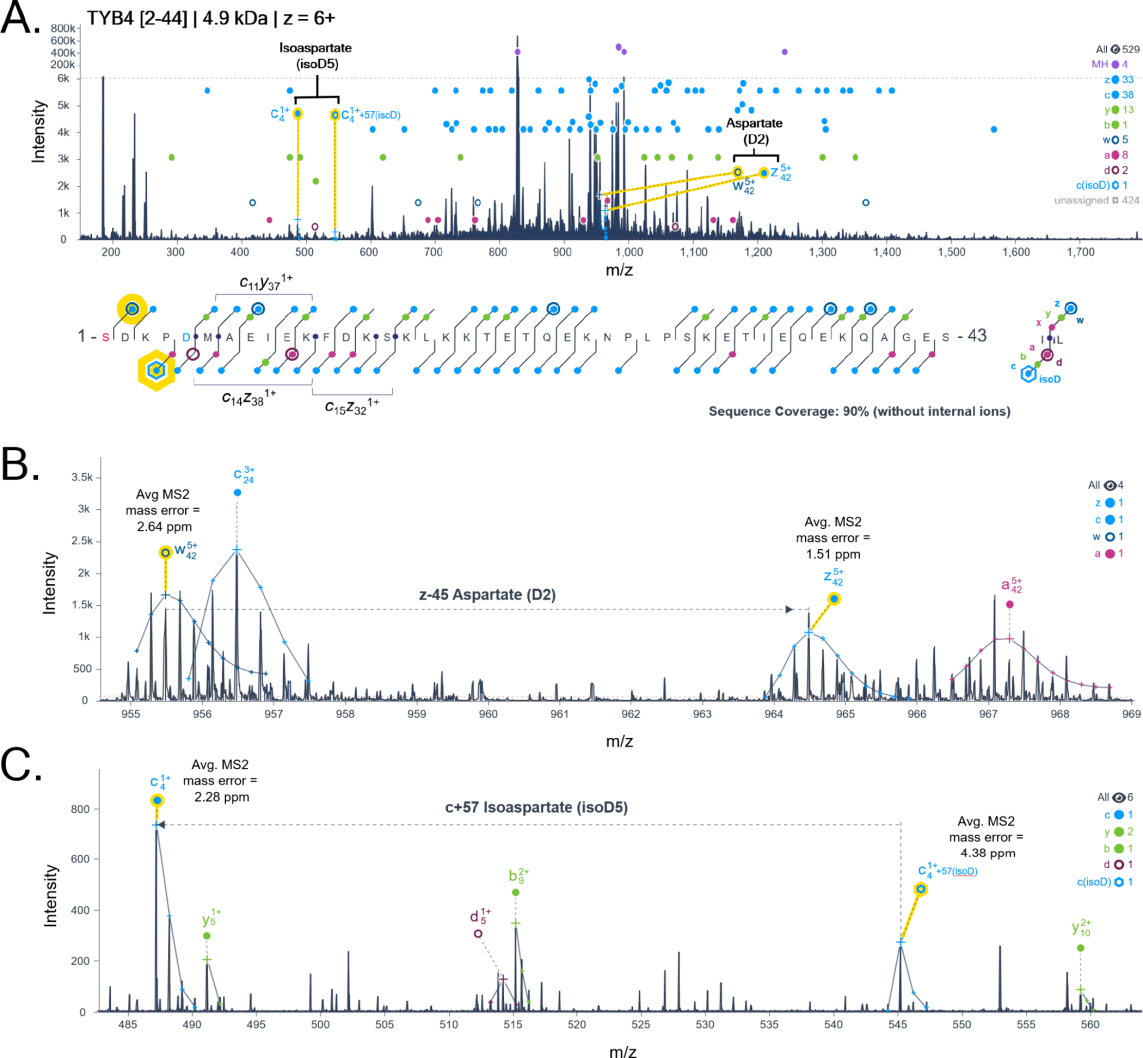
Accurate differentiation of aspartate and isoaspartate residues in the identified MS/MS spectra and sequence coverage map of a thymosin beta-4 [2-44] proteoform (A) from an acetone-precipitated intact protein sample from human brain. Within this proteoform, aspartate at position 2 was confirmed through a paired w-ion fragment (z −45 Da) with the respective intact z-ion (B). Isoaspartate was confirmed at position 5 through detection of +57 Da mass on the c- ion (C). Red-colored letters in sequence coverage map indicate acetylation. Blue-colored letters in sequence coverage map indicate isoaspartate. Yellow highlights indicate fragments of interest. Sequence position is relative to the sequence presented and not the full-length protein. Charge state included in the label indicates what was identified and used for analysis.

Another benefit of utilizing electron-associated dissociation is that the fragmentation modality allows for accurate differentiation between leucine and isoleucine residues through side-chain fragmentations.^37–39^ Leucine resides can be identified identification of complementary *z*- ion to *w*- ion with a mass shift of −43 Da as the isobutyl side chain losses a radical isopropyl via electron-associated fragmentation. The *sec*-butyl side chain of isoleucine has mass shift of −29 Da due to loss of a radical methyl due to fragmentation. In the proteoforms we identified, we have an example of sequencing and differentiation leucine and isoleucine in the same proteoform within 5 residues from one another, in ubiquitin proteoform originating from polyubiquitin-b [1–74] (Figure 5A). In this case, we were able to accurately sequence L67 (Figure 5B) and I61 (Figure 5C) both through their respective side chain fragmentation. Accurate sequencing as offered through ECD would provide a significant advantage for de novo sequencing for identifying novel peptide and proteoforms and overcoming the current database limitations we rely on for both untargeted database searches. Here, we can bypass the requirement of needing to have the nucleotide sequences to determine whether leucine or isoleucine was identified *a priori*. Traditional collision-based do not offer this level information on isomers unless complemented with ion mobility^40,41^ and even then the leucine and isoleucine ambiguity persists.

**Figure 5:**
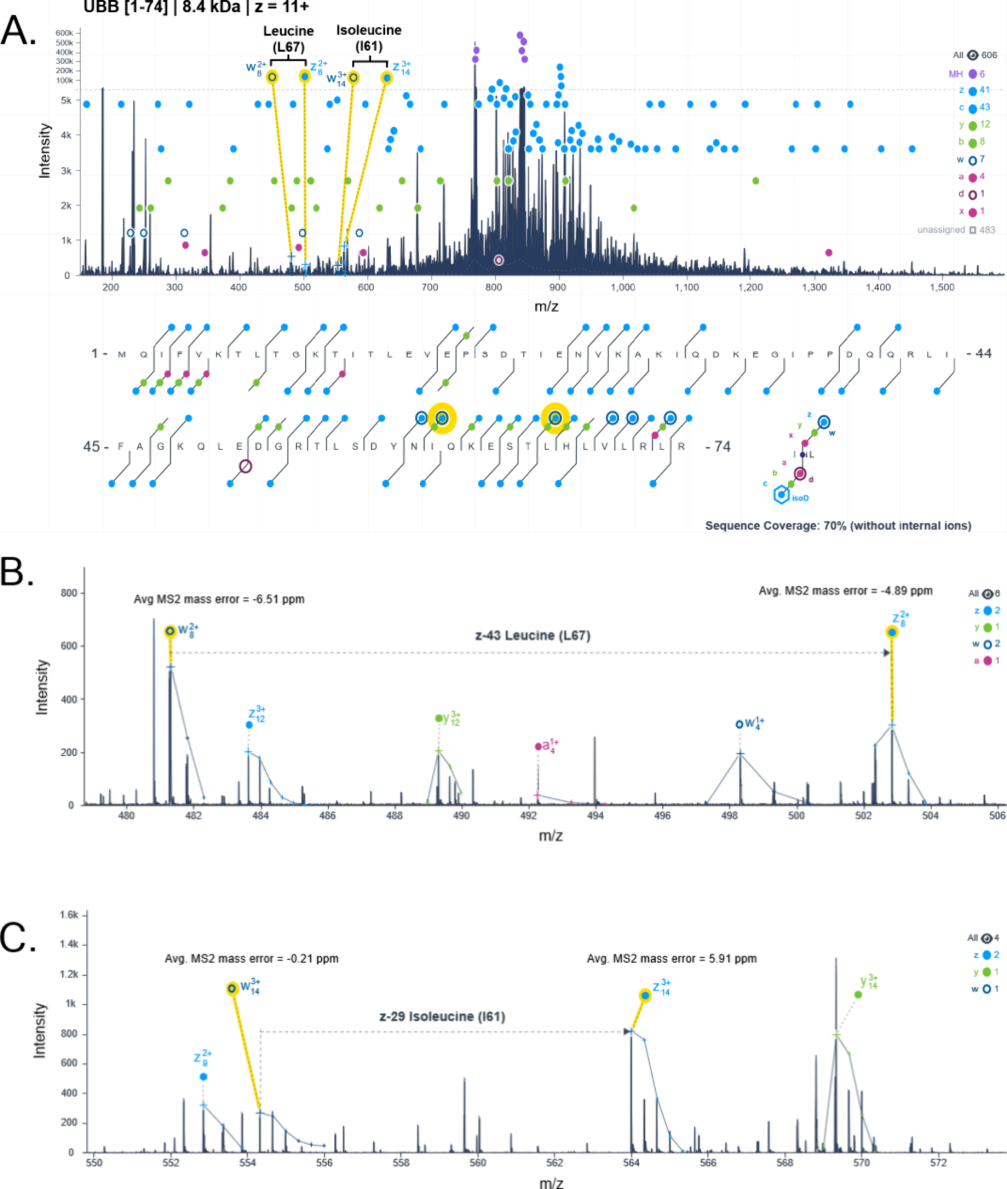
Accurate differentiation of leucine and isoleucine residues in the identified MS/MS spectra and sequence coverage map of a polyubiquitin-b [1-74] proteoform (A) from an acetone-precipitated intact protein sample from human brain. Within this proteoform, leucine at position 67 was confirmed through paired intact z- ion and side chain fragmented w- ion (−43 Da) (B). Isoleucine at position 61 was confirmed through paired intact z- ion and side chain fragmented w- ion (−29 Da) (C). Sequence position is relative to the sequence presented and not the full-length protein. Charge state included in label indicates what was identified and used for analysis.

One of the main limitations of this study is that lack of detection of proteins above 17 kDa. We attribute this to difficulties with resuspending acetone-precipitated pellet into water, which is anticipated for acetone-based protein purification^42^ and typically requires harsh conditions like 6 M urea or detergents like SDS. Our aim was to generate a sample containing multiple proteins to evaluate if intact top-down proteomics (TDP) via ECD, coupled with LC-based sample introduction, could be viable. Chromatography of a full proteome without prior fractionation of simplification results in crowded spectra lacking defining features that could be automatically detected in a DDA format. While the sample was not ideal, it served this purpose: we successfully identified several small proteins and peptides across a chromatographic gradient using ECD for proteoform sequencing. And even identified difficult novel isoaspartate PTM for TYB4. Although our sample was limited to 17 kDa, we would like to highlight that ECD has strong application to proteins above 17 kDa. Greisch et al. demonstrated that TDP ECD of ∼MDa immunoglobulin (Ig) complexes resulted in efficient fragmentation of the sequence tags of the regions of the IgMs, which importantly includes variable complementarity-determining regions.^43^ Here a modified ultrahigh mass range Q-Exactive Orbitrap equipped with a modified collision and ECD cell made by e-MSion. Their findings and ours demonstrated the low-energy ECD via the e-MSion ECD cell can perform ECD on analytes ranging from peptides up to MDa protein complexes.

In a similar approach to what we have presented, LC-ECD-FT-ICR was previous demonstration of the utility of LC coupled to ECD for DDA BUP for the sequencing of tryptic peptides on chromatographic time scales.^44^ Cooper et al. showed that ECD was compatible with LC-MS/MS DDA workflows and suitable for high-throughput proteomics. For TDP, Parks et al. utilized at hybrid linear ion trap Fourier transform instrument for high resolution online DDA LC-MS/MS of proteoforms, where they found 22 proteins and up to 231 proteoforms from a yeast whole-cell lysate.^45^ Our findings are analogous, except we employ a Q-TOF platform and TDP strategy to identify intact proteoforms. As such, our work represents an early demonstration of the viability of LC-Q-ECD-TOF instrumentation for TDP applications.

In many current LC-MS/MS TDP workflows using electron-based dissociation, Orbitrap-based platforms equipped with ETD are commonly employed for both BUP and TDP. Another limitation of our study is the relatively low proteome coverage achieved, which again we attribute primarily to the low complexity of our sample due to resuspension efficiency of acetone-precipitated proteins. Consequently, our data set was enriched for a limited number of peptides and smaller proteoforms (Figure 3A). For context, Dupré et al. utilized a hybrid electron transfer higher-energy dissociation (EThcD)-based method due to increased sequence coverage and higher identification scores as compared to HCD to identify more than 500 proteoforms spanning 220 proteins within *Escherichia coli* samples.^46^ Fulcher et al., preformed TDP on human brain tissue with sample fractionation, and was able to identify 11,782 proteoforms over 1,212 proteins in 103 samples utilizing a hybrid Orbitrap and linear ion trap instrument, and CID MS/MS method for proteoform identification.^47^ Their study demonstrates that the field has reached over 1,000 proteins identified in human brain using TDP.

ECD has been explored in an Orbitrap-based instrument by Fort et al. where ECD was able to outperform HCD-only methods with sequence coverage of >60% for intact proteins.^48^ The benefit of ECD as compared to ETD is reduced reaction time needed to facilitate efficient fragmentation. Comparing ubiquitin results, Fort et al. found sequence coverage of 80% with static spray being the sample introduction method, while we observed 17–70% sequence coverage for the ubiquitin proteoforms using LC for sample introduction. This highlights the efficiency of ECD for sequencing small proteins on chromatographic time scales with only a few seconds of MS2 accumulation or less compared to ∼1 min acquisition with static nano spray.

Future work will focus on optimizing LC conditions, gradient profiles, sample preparation, DDA parameters, and ECD tuning to increase proteome coverage using LC-Q-TOF platforms. Although not explored here, hybrid fragmentation strategies such as electron capture collision-induced dissociation (ECciD) may further enhance sequence coverage. In this approach, ECD fragments generated in the e-MSion cell could be further dissociated using the standard Agilent CID cell in series. However, the utility of ECciD for labile PTMs remains uncertain due to the ergodic nature of CID. Fulcher et al. characterized up to 49 Aβ proteoforms^47^ and we have previously shown that 80% of Aβ from Alzheimer’s brain tissue contains isoaspartate.^34^ A future direction for us to extract and characterize Aβ proteoforms, including isoaspartate, directly from human tissue by leveraging our ECD workflow presented here.

The overall goal of this study was to evaluate the feasibility of ECD for TDP on chromatographic time scales. We demonstrated that ECD enables high sequence coverage for individual proteins introduced via LC, achieving 95% sequence coverage for full-length Aβ40. We also showed that ECD-only DDA methods are capable of identifying multiple peptides and small proteoforms over chromatographic gradients, with sequence coverage ranging from 7–90%, depending on peptide and proteoform size and composition. In the same experiment, we demonstrated the utility of ECD for isobaric species identification for accurate differentiation of isoleucine versus leucine and aspartate versus isoaspartate through fragmentation. Our findings validate ECD as a viable dissociation technique for LC-TDP workflows and motivate continued method development using this approach, which are accessible on Agilent Q-TOF instruments via the Agilent ExD cell.

## Conclusions

We show that an LC-Q-TOF instrument can be expanded to include ECD fragmentation and that ECD fragmentation can operate with LC time scales. Significantly, we demonstrated that the efficiency of ECD is sufficient for the top-down sequencing of intact proteins for proteoform identification and real-world application of aspartate and isoaspartate and leucine and isoleucine differentiation within a sample human brain tissue. This study marks a significant advance for the use of LC-Q-TOF and ECD technologies for discovery TDP.

## Supplementary Material



## Data Availability

Analysis files from the presented work, TopPIC and ExDViewer, are available upon request to the corresponding author.

## Notes

Agilent 6550 instrument method for top-down sequencing of amyloid beta 1-40, Agilent 6550 instrument method for data-dependent acquisition-based top-down proteomics of intact proteins extracted from human brain tissues, and sample processing workflow for extraction of intact proteins from human brain tissues (PDF)
