# Supplementary material for: Electron Capture Dissociation for Discovery Top-Down Proteomics of Peptides and Small Proteins on Chromatographic Time Scales
Source: J Am Soc Mass Spectrom. 2025 Aug 28;36(10):2079–93. doi: 10.1021/jasms.5c00116 (PMC12492394; doi:10.1021/jasms.5c00116)
Supplement: Supplementary file 1 [file js5c00116_si_001.pdf]

Supporting Information

Electron capture dissociation for discovery top-down proteomics of peptides and small proteins on chromatographic timescales

Lester S. Manly[1,2], Anne M. Roberts[1], Joseph S. Beckman[3], Blaine R. Roberts*[1,2,4]

[1]Department of Biochemistry, Emory University School of Medicine, Atlanta, Georgia, 30322, USA

[2]Molecular and Systems Pharmacology Graduate Program, Graduate Division of Biological and Biomedical Sciences, Laney Graduate School, Emory University, Atlanta, Georgia, 30322, USA

[3]Linus Pauling Institute, Oregon State University, Corvallis, Oregon, 97330, USA

[4]Department of Neurology, Emory University School of Medicine, Atlanta, Georgia, 30322, USA

Corresponding Author

Blaine R. Roberts

blaine.roberts@emory.edu

Supplementary Information

S-1: Agilent 6550 instrument method for top-down sequencing amyloid beta 1-40

S-2: Agilent 6550 instrument method for data-dependent acquisition-based top-down proteomics of intact proteins extract from human brain tissues

S-3: Sample processing workflow for extracting intact proteins from human brain tissues

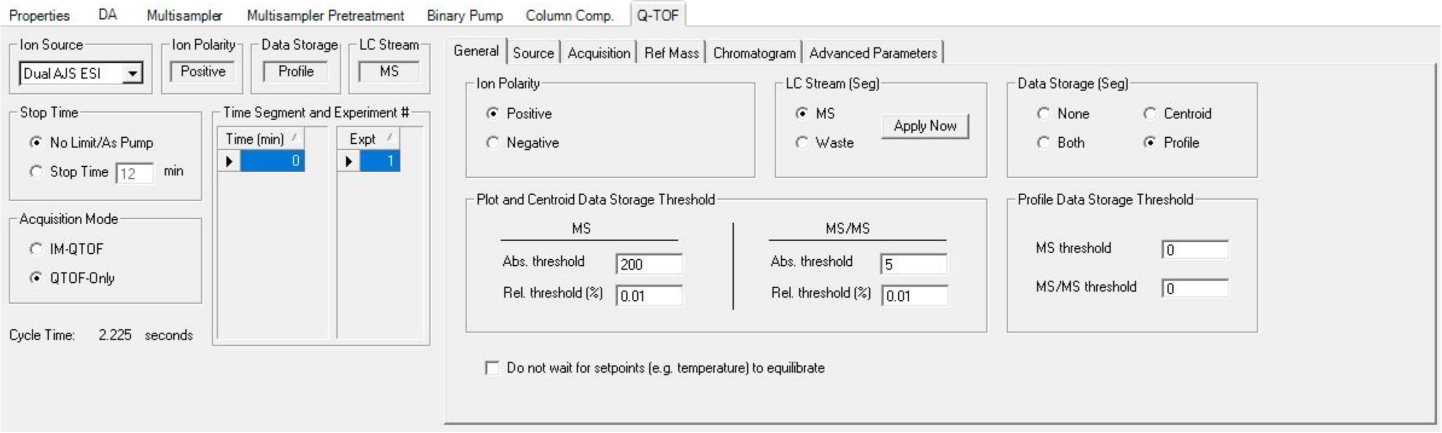
Properties DA Multisampler Multisampler Pretreatment Binary Pump Column Comp. Q-TOF
Ion Source Dual AJS ESI
Ion Polarity Positive
Data Storage Profile
LC Stream MS
Stop Time
No Limit/As Pump
Stop Time 12 min
Time Segment and Experiment #
Time (min) 0
Expt 1
Acquisition Mode
IM-QTOF
QTOF-Only
Cycle Time: 2.225 seconds
General Source Acquisition Ref Mass Chromatogram Advanced Parameters
Ion Polarity
Positive
Negative
LC Stream (Seg)
MS
Waste
Apply Now
Data Storage (Seg)
None
Centroid
Both
Profile
Plot and Centroid Data Storage Threshold
MS
Abs. threshold 200
Rel. threshold (%) 0.01
MS/MS
Abs. threshold 5
Rel. threshold (%) 0.01
Profile Data Storage Threshold
MS threshold 0
MS/MS threshold 0
Do not wait for setpoints (e.g. temperature) to equilibrate

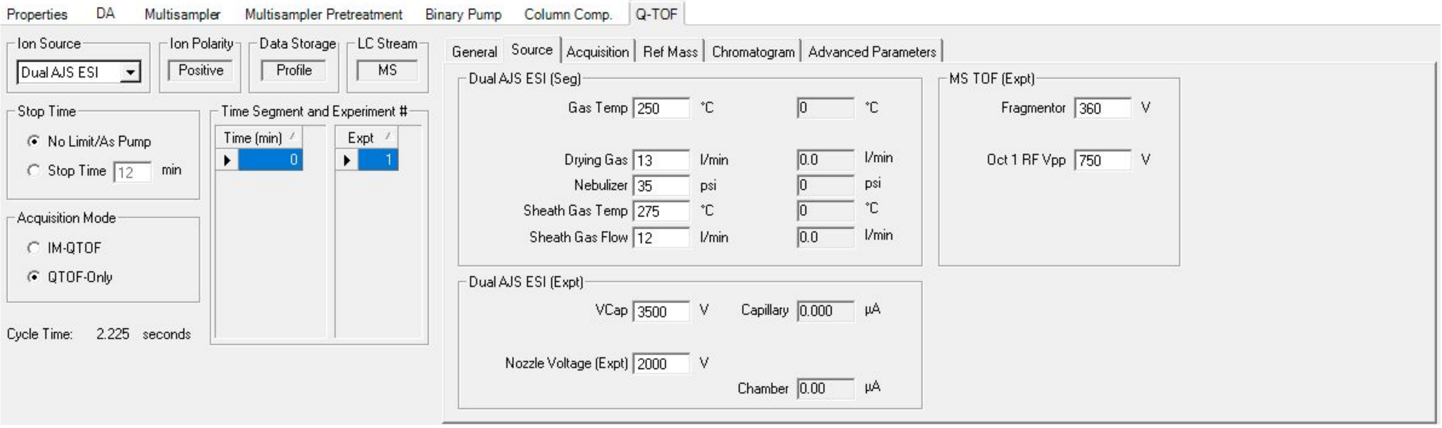
Properties DA Multisampler Multisampler Pretreatment Binary Pump Column Comp. Q-TOF
Ion Source Dual AJS ESI
Ion Polarity Positive
Data Storage Profile
LC Stream MS
Stop Time
No Limit/As Pump
Stop Time 12 min
Time Segment and Experiment #
Time (min) 0
Expt 1
Acquisition Mode
IM-QTOF
QTOF-Only
Cycle Time: 2.225 seconds
General Source Acquisition Ref Mass Chromatogram Advanced Parameters
Dual AJS ESI (Seg)
Gas Temp 250 °C 0 °C
Drying Gas 13 l/min 0.0 l/min
Nebulizer 35 psi 0 psi
Sheath Gas Temp 275 °C 0 °C
Sheath Gas Flow 12 l/min 0.0 l/min
MS TOF (Expt)
Fragmentor 360 V
Oct 1 RF Vpp 750 V
Dual AJS ESI (Expt)
VCap 3500 V
Capillary 0.000 µA
Nozzle Voltage (Expt) 2000 V
Chamber 0.00 µA

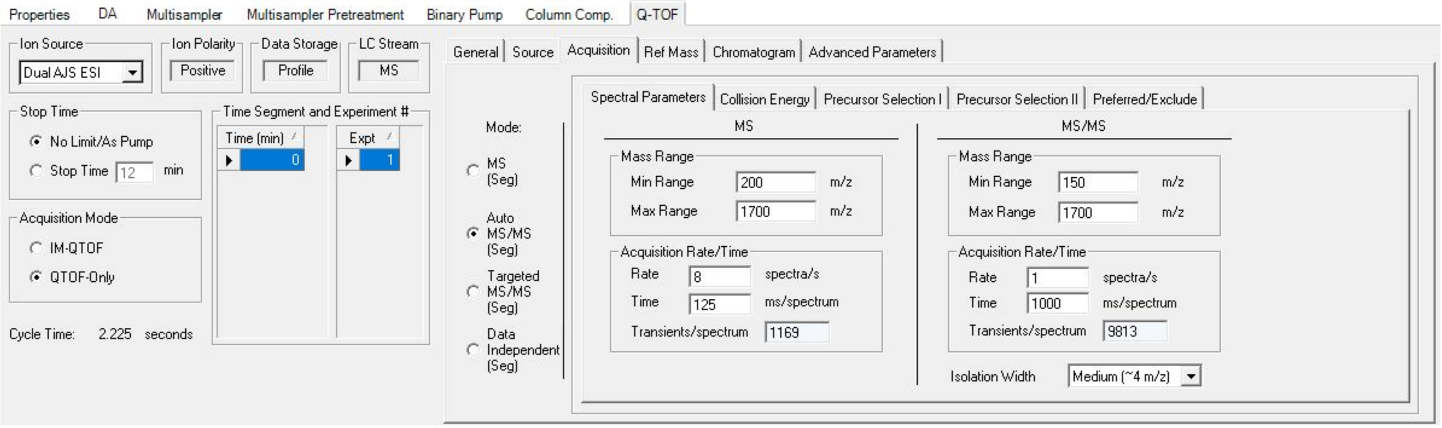
Properties DA Multisampler Multisampler Pretreatment Binary Pump Column Comp. Q-TOF
Ion Source Dual AJS ESI
Ion Polarity Positive
Data Storage Profile
LC Stream MS
Stop Time
No Limit/As Pump
Stop Time 12 min
Time Segment and Experiment #
Time (min) 0
Expt 1
Acquisition Mode
IM-QTOF
QTOF-Only
Cycle Time: 2.225 seconds
General Source Acquisition Ref Mass Chromatogram Advanced Parameters
Mode:
MS (Seg)
Auto MS/MS (Seg)
Targeted MS/MS (Seg)
Data Independent (Seg)
Spectral Parameters Collision Energy Precursor Selection I Precursor Selection II Preferred/Exclude
MS
Mass Range
Min Range 200 m/z
Max Range 1700 m/z
Acquisition Rate/Time
Rate 8 spectra/s
Time 125 ms/spectrum
Transients/spectrum 1169
MS/MS
Mass Range
Min Range 150 m/z
Max Range 1700 m/z
Acquisition Rate/Time
Rate 1 spectra/s
Time 1000 ms/spectrum
Transients/spectrum 9813
Isolation Width Medium (~4 m/z)

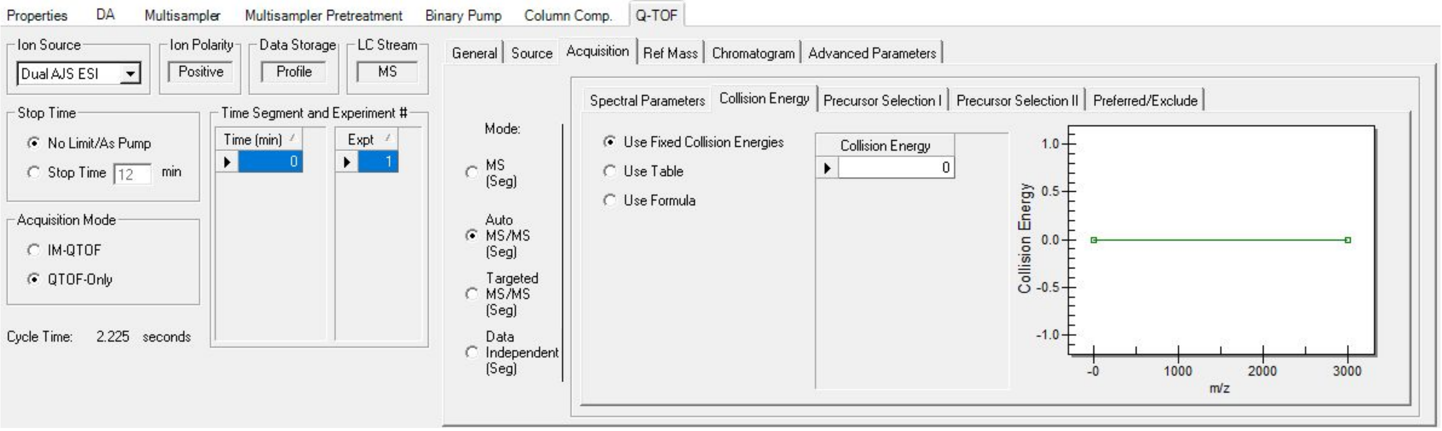
Properties DA Multisampler Multisampler Pretreatment Binary Pump Column Comp. Q-TOF
Ion Source Dual AJS ESI
Ion Polarity Positive
Data Storage Profile
LC Stream MS
Stop Time
No Limit/As Pump
Stop Time 12 min
Time Segment and Experiment #
Time (min) 0
Expt 1
Acquisition Mode
IM-QTOF
QTOF-Only
Cycle Time: 2.225 seconds
General Source Acquisition Ref Mass Chromatogram Advanced Parameters
Mode:
MS (Seg)
Auto MS/MS (Seg)
Targeted MS/MS (Seg)
Data Independent (Seg)
Spectral Parameters Collision Energy Precursor Selection I Precursor Selection II Preferred/Exclude
Use Fixed Collision Energies
Use Table
Use Formula
Collision Energy 0
Collision Energy
1.0
0.5
0.0
-0.5
-1.0
-0
1000
2000
3000
m/z

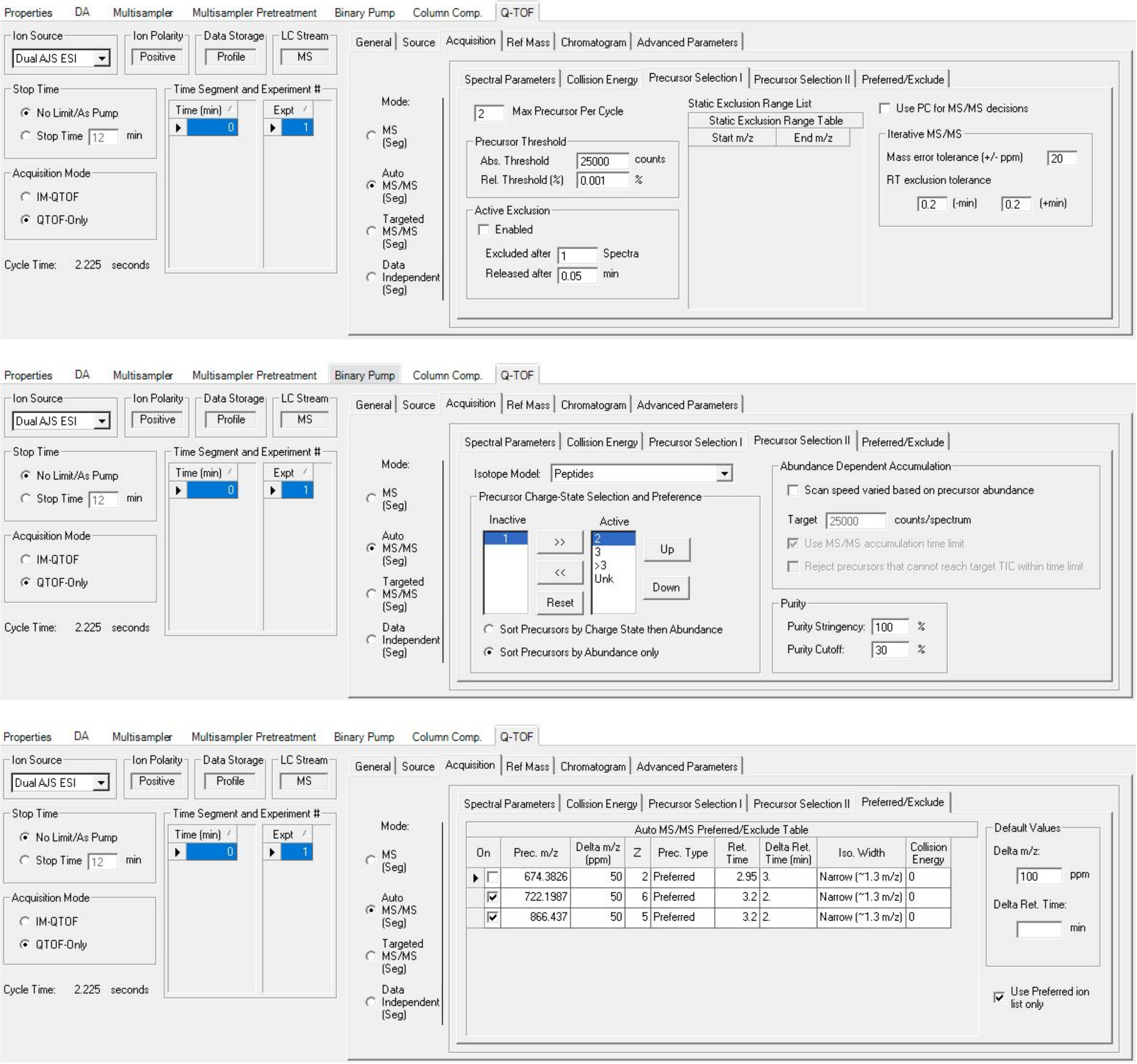

Supplemental Figure 1. Agilent 6550 source and data-dependent acquisition settings for top-down proteomics using reverse-phase liquid chromatography and electron capture dissociation fragmentation of synthetic full-length amyloid beta 1-40.

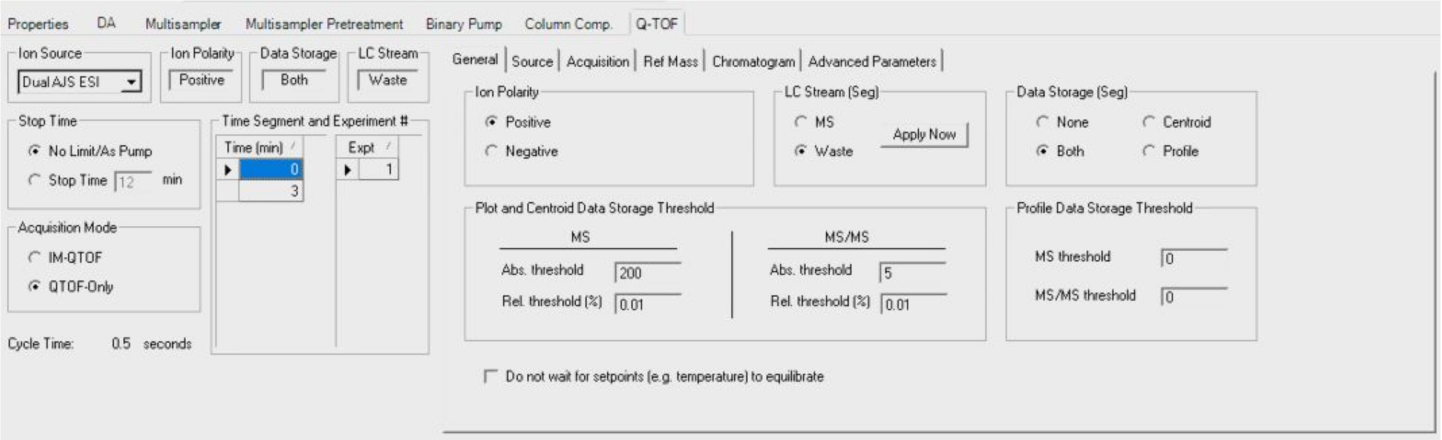
Properties DA Multisampler Multisampler Pretreatment Binary Pump Column Comp. Q-TOF
Ion Source
Dual AJS ESI
Ion Polarity
Positive
Data Storage
Both
LC Stream
Waste
Stop Time
No Limit/As Pump
Stop Time 12 min
Time Segment and Experiment #
Time (min)
0
3
Expt
1
Acquisition Mode
IM-QTOF
QTOF-Only
Cycle Time: 0.5 seconds
General Source Acquisition Ref Mass Chromatogram Advanced Parameters
Ion Polarity
Positive
Negative
LC Stream (Seg)
MS
Waste
Apply Now
Data Storage (Seg)
None
Centroid
Both
Profile
Plot and Centroid Data Storage Threshold
MS
Abs. threshold 200
Rel. threshold (%) 0.01
MS/MS
Abs. threshold 5
Rel. threshold (%) 0.01
Profile Data Storage Threshold
MS threshold 0
MS/MS threshold 0
Do not wait for setpoints (e.g. temperature) to equilibrate

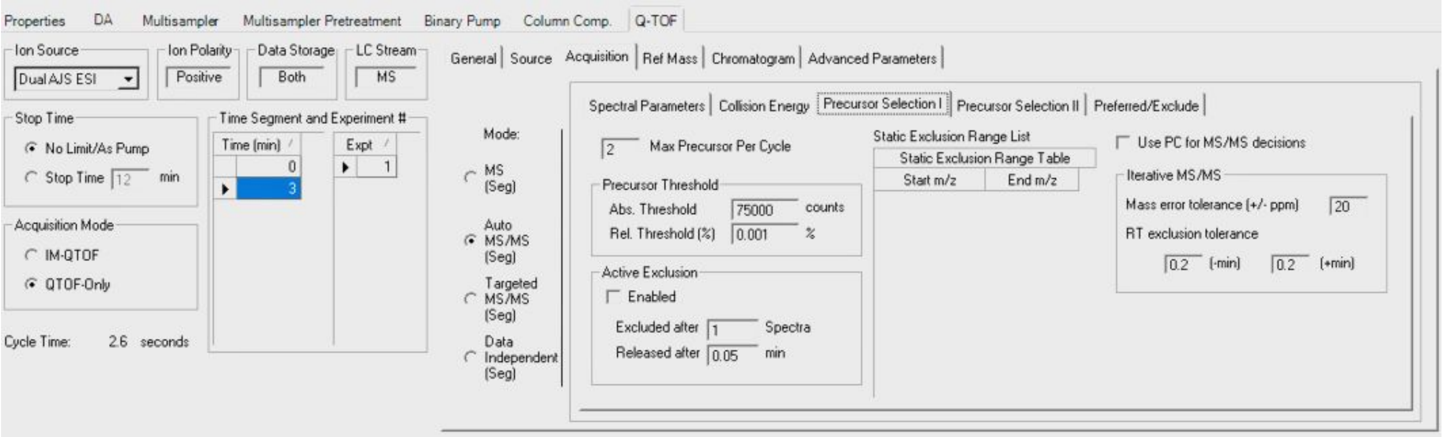
Properties DA Multisampler Multisampler Pretreatment Binary Pump Column Comp. Q-TOF
Ion Source
Dual AJS ESI
Ion Polarity
Positive
Data Storage
Both
LC Stream
MS
Stop Time
No Limit/As Pump
Stop Time 12 min
Time Segment and Experiment #
Time (min)
0
3
Expt
1
Acquisition Mode
IM-QTOF
QTOF-Only
Cycle Time: 2.6 seconds
General Source Acquisition Ref Mass Chromatogram Advanced Parameters
Mode:
MS (Seg)
Auto MS/MS (Seg)
Targeted MS/MS (Seg)
Data Independent (Seg)
Spectral Parameters Collision Energy Precursor Selection I Precursor Selection II Preferred/Exclude
2 Max Precursor Per Cycle
Precursor Threshold
Abs. Threshold 75000 counts
Rel. Threshold (%) 0.001 %
Active Exclusion
Enabled
Excluded after 1 Spectra
Released after 0.05 min
Static Exclusion Range List
Static Exclusion Range Table
Start m/z
End m/z
Use PC for MS/MS decisions
Iterative MS/MS
Mass error tolerance (+/- ppm) 20
RT exclusion tolerance
0.2 (-min) 0.2 (+min)

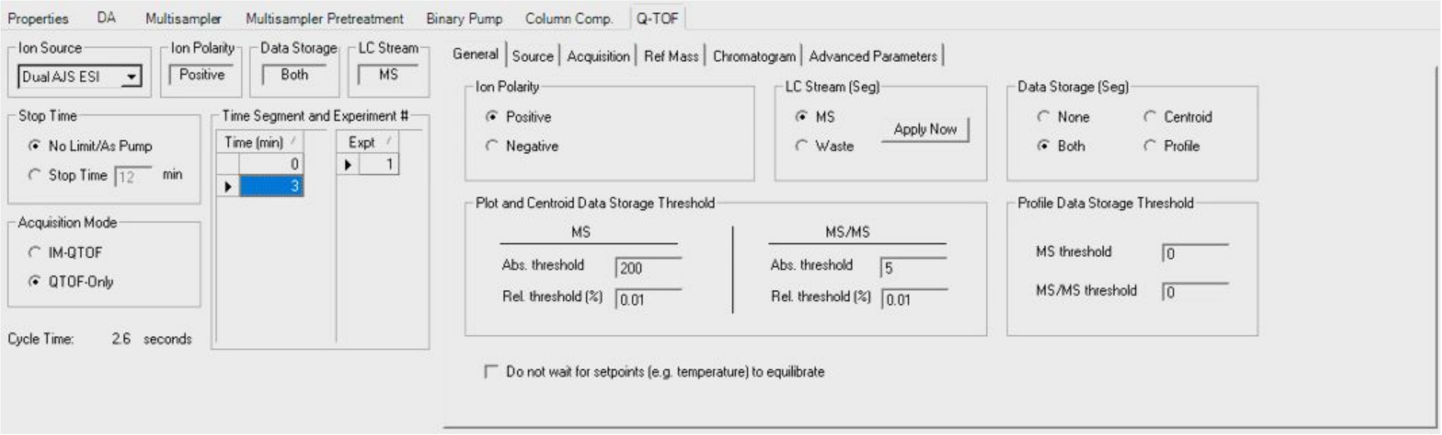
Properties DA Multisampler Multisampler Pretreatment Binary Pump Column Comp. Q-TOF
Ion Source
Dual AJS ESI
Ion Polarity
Positive
Data Storage
Both
LC Stream
MS
Stop Time
No Limit/As Pump
Stop Time 12 min
Time Segment and Experiment #
Time (min)
0
3
Expt
1
Acquisition Mode
IM-QTOF
QTOF-Only
Cycle Time: 2.6 seconds
General Source Acquisition Ref Mass Chromatogram Advanced Parameters
Ion Polarity
Positive
Negative
LC Stream (Seg)
MS
Waste
Apply Now
Data Storage (Seg)
None
Centroid
Both
Profile
Plot and Centroid Data Storage Threshold
MS
Abs. threshold 200
Rel. threshold (%) 0.01
MS/MS
Abs. threshold 5
Rel. threshold (%) 0.01
Profile Data Storage Threshold
MS threshold 0
MS/MS threshold 0
Do not wait for setpoints (e.g. temperature) to equilibrate

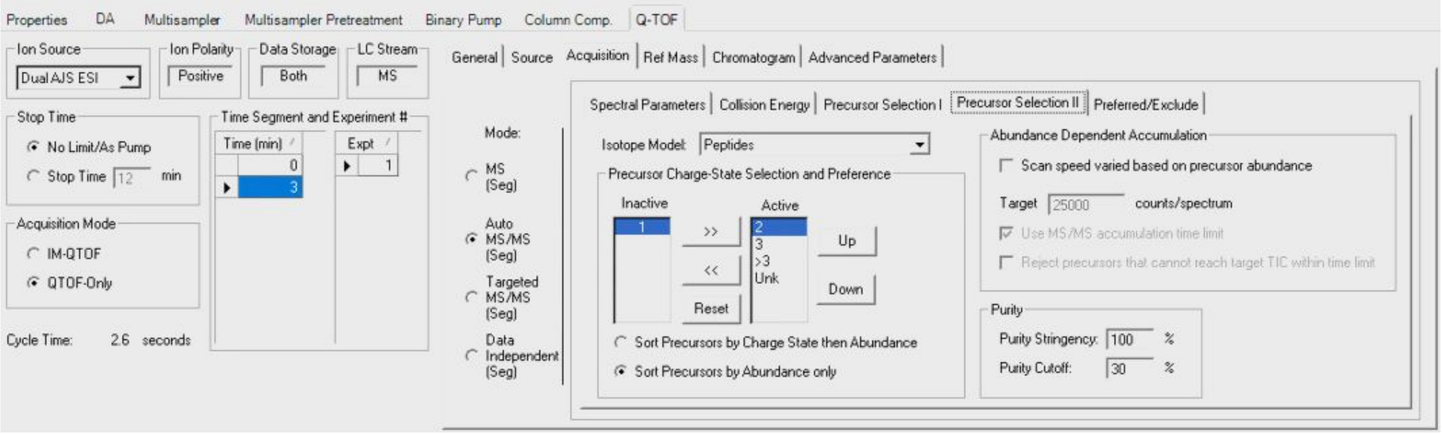
Properties DA Multisampler Multisampler Pretreatment Binary Pump Column Comp. Q-TOF
Ion Source
Dual AJS ESI
Ion Polarity
Positive
Data Storage
Both
LC Stream
MS
Stop Time
No Limit/As Pump
Stop Time 12 min
Time Segment and Experiment #
Time (min)
0
3
Expt
1
Acquisition Mode
IM-QTOF
QTOF-Only
Cycle Time: 2.6 seconds
General Source Acquisition Ref Mass Chromatogram Advanced Parameters
Mode:
MS (Seg)
Auto MS/MS (Seg)
Targeted MS/MS (Seg)
Data Independent (Seg)
Spectral Parameters Collision Energy Precursor Selection I Precursor Selection II Preferred/Exclude
Isotope Model: Peptides
Precursor Charge-State Selection and Preference
Inactive
1
Active
2
3
>3
Unk
>>
<<
Reset
Up
Down
Sort Precursors by Charge State then Abundance
Sort Precursors by Abundance only
Abundance Dependent Accumulation
Scan speed varied based on precursor abundance
Target 25000 counts/spectrum
Use MS/MS accumulation time limit
Reject precursors that cannot reach target TIC within time limit
Purity
Purity Stringency: 100 %
Purity Cutoff: 30 %

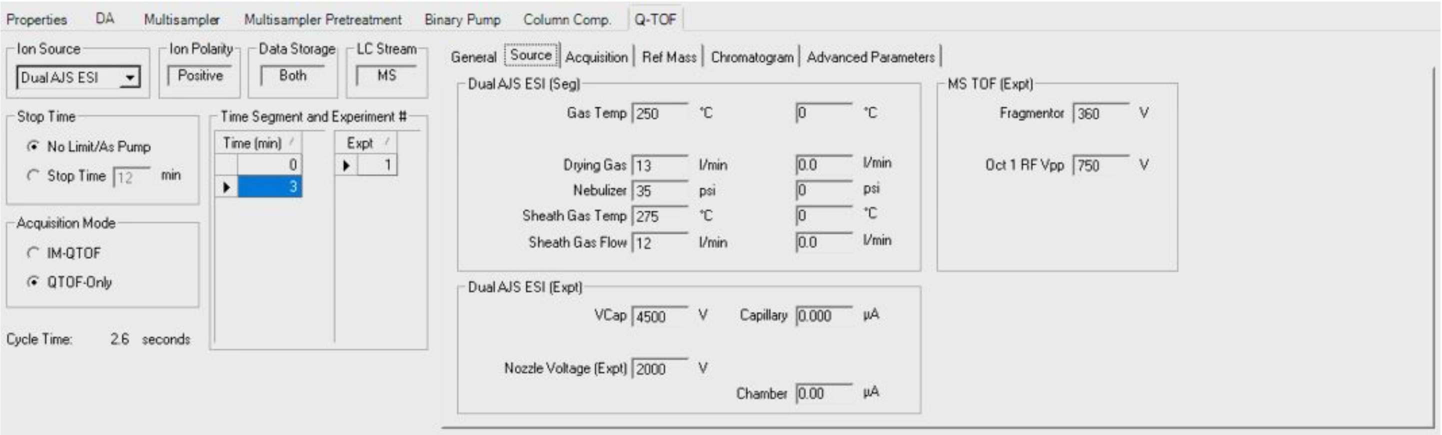
Properties DA Multisampler Multisampler Pretreatment Binary Pump Column Comp. Q-TOF
Ion Source Dual AJS ESI
Ion Polarity Positive
Data Storage Both
LC Stream MS
Stop Time No Limit/As Pump Stop Time 12 min
Time Segment and Experiment #
Time (min) 0 3
Expt 1
Acquisition Mode IM-QTOF QTOF-Only
Cycle Time: 2.6 seconds
General Source Acquisition Ref Mass Chromatogram Advanced Parameters
Dual AJS ESI (Seg)
Gas Temp 250 °C 0 °C
Drying Gas 13 l/min 0.0 l/min
Nebulizer 35 psi 0 psi
Sheath Gas Temp 275 °C 0 °C
Sheath Gas Flow 12 l/min 0.0 l/min
MS TOF (Expt)
Fragmentor 360 V
Oct 1 RF Vpp 750 V
Dual AJS ESI (Expt)
VCap 4500 V Capillary 0.000 µA
Nozzle Voltage (Expt) 2000 V
Chamber 0.00 µA

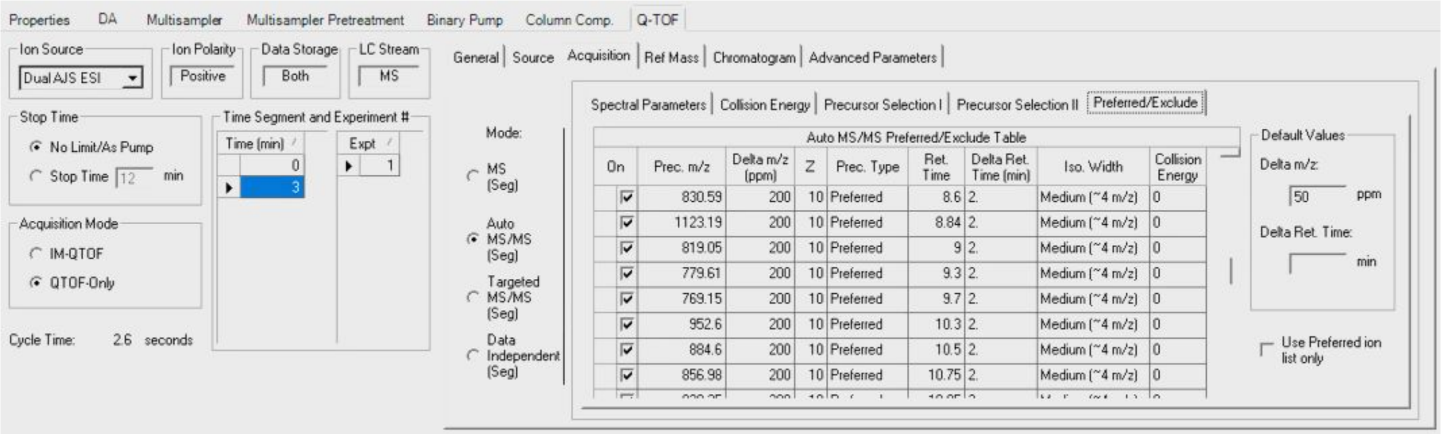
Properties DA Multisampler Multisampler Pretreatment Binary Pump Column Comp. Q-TOF
Ion Source Dual AJS ESI
Ion Polarity Positive
Data Storage Both
LC Stream MS
Stop Time No Limit/As Pump Stop Time 12 min
Time Segment and Experiment #
Time (min) 0 3
Expt 1
Acquisition Mode IM-QTOF QTOF-Only
Cycle Time: 2.6 seconds
General Source Acquisition Ref Mass Chromatogram Advanced Parameters
Spectral Parameters Collision Energy Precursor Selection I Precursor Selection II Preferred/Exclude
Mode: MS (Seg) Auto MS/MS (Seg) Targeted MS/MS (Seg) Data Independent (Seg)
Auto MS/MS Preferred/Exclude Table
On Prec. m/z Delta m/z (ppm) Z Prec. Type Ret. Time Delta Ret. Time (min) Iso. Width Collision Energy
830.59 200 10 Preferred 8.6 2. Medium (~4 m/z) 0
1123.19 200 10 Preferred 8.84 2. Medium (~4 m/z) 0
819.05 200 10 Preferred 9 2. Medium (~4 m/z) 0
779.61 200 10 Preferred 9.3 2. Medium (~4 m/z) 0
769.15 200 10 Preferred 9.7 2. Medium (~4 m/z) 0
952.6 200 10 Preferred 10.3 2. Medium (~4 m/z) 0
884.6 200 10 Preferred 10.5 2. Medium (~4 m/z) 0
856.98 200 10 Preferred 10.75 2. Medium (~4 m/z) 0
Default Values
Delta m/z: 50 ppm
Delta Ret. Time: min
Use Preferred ion list only

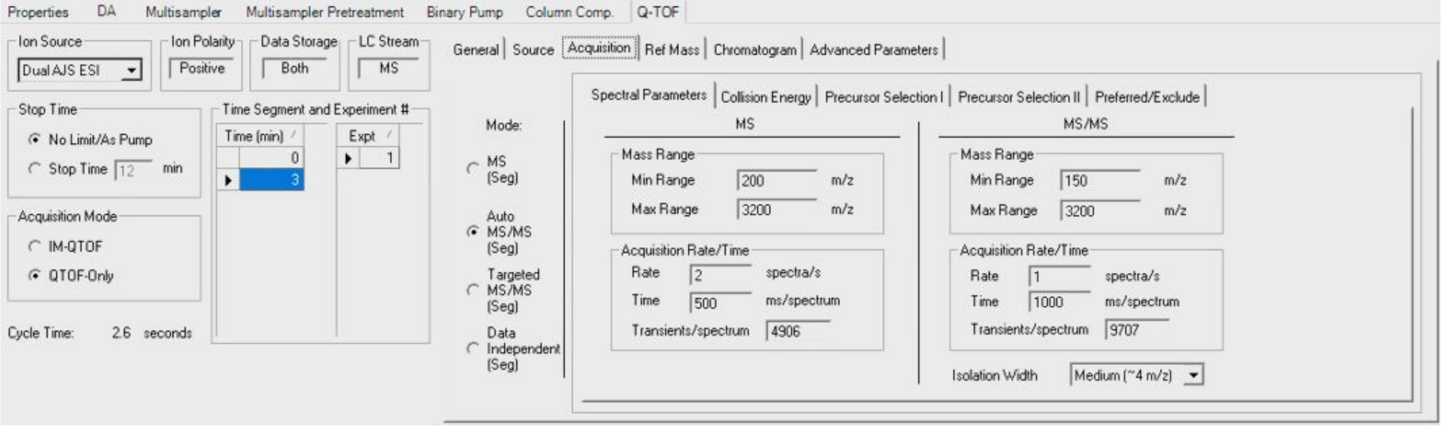
Properties DA Multisampler Multisampler Pretreatment Binary Pump Column Comp. Q-TOF
Ion Source Dual AJS ESI
Ion Polarity Positive
Data Storage Both
LC Stream MS
Stop Time No Limit/As Pump Stop Time 12 min
Time Segment and Experiment #
Time (min) 0 3
Expt 1
Acquisition Mode IM-QTOF QTOF-Only
Cycle Time: 2.6 seconds
General Source Acquisition Ref Mass Chromatogram Advanced Parameters
Spectral Parameters Collision Energy Precursor Selection I Precursor Selection II Preferred/Exclude
Mode: MS (Seg) Auto MS/MS (Seg) Targeted MS/MS (Seg) Data Independent (Seg)
MS
Mass Range
Min Range 200 m/z
Max Range 3200 m/z
Acquisition Rate/Time
Rate 2 spectra/s
Time 500 ms/spectrum
Transients/spectrum 4906
MS/MS
Mass Range
Min Range 150 m/z
Max Range 3200 m/z
Acquisition Rate/Time
Rate 1 spectra/s
Time 1000 ms/spectrum
Transients/spectrum 9707
Isolation Width Medium (~4 m/z)

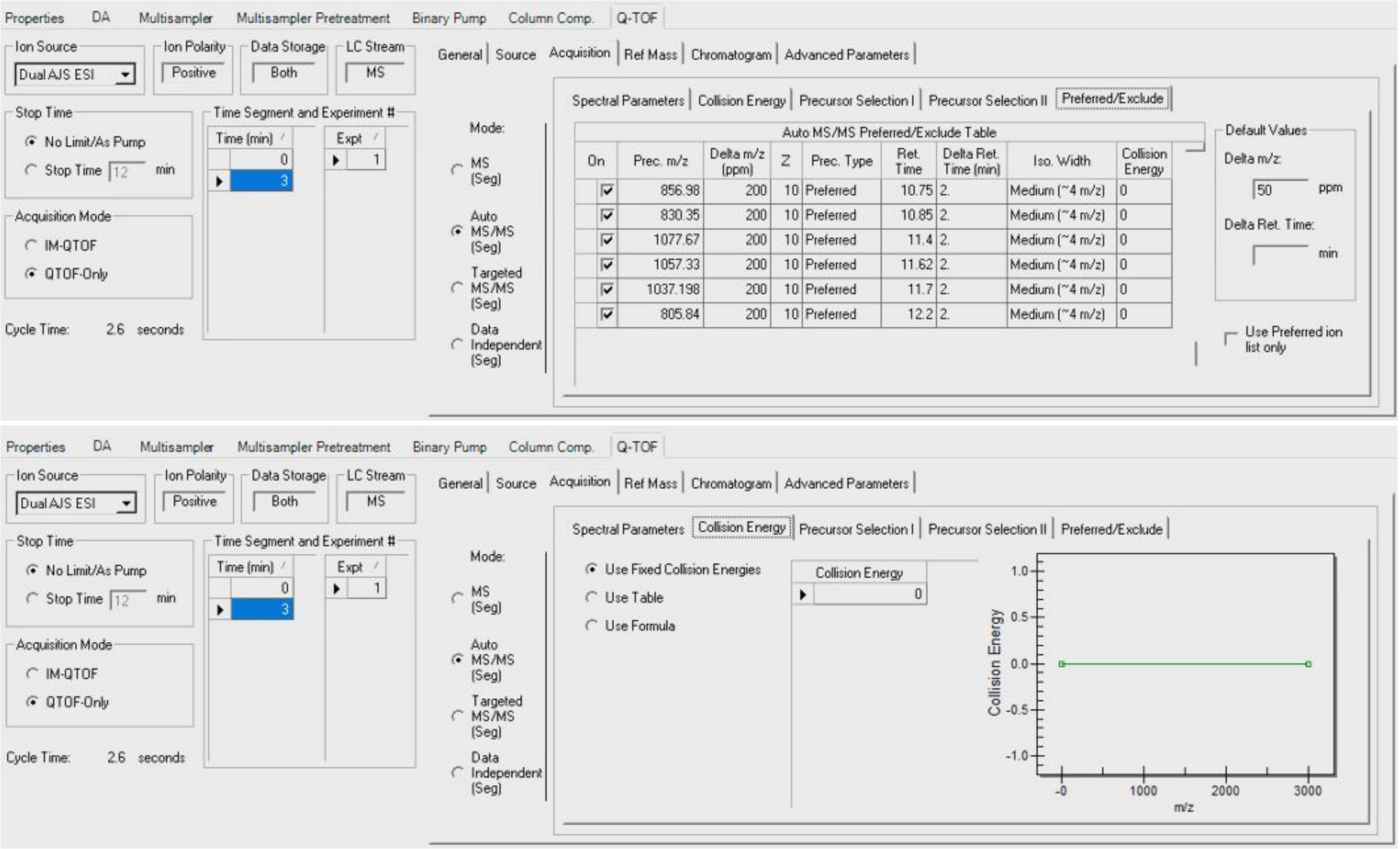

Supplemental Figure 2 settings, top panel (Auto MS/MS Preferred/Exclude Table):

On	Prec. m/z	Delta m/z (ppm)	Z	Prec. Type	Ret. Time	Delta Ret. Time (min)	Iso. Width	Collision Energy
☑	856.98	200	10	Preferred	10.75	2.	Medium (~4 m/z)	0
☑	830.35	200	10	Preferred	10.85	2.	Medium (~4 m/z)	0
☑	1077.67	200	10	Preferred	11.4	2.	Medium (~4 m/z)	0
☑	1057.33	200	10	Preferred	11.62	2.	Medium (~4 m/z)	0
☑	1037.198	200	10	Preferred	11.7	2.	Medium (~4 m/z)	0
☑	805.84	200	10	Preferred	12.2	2.	Medium (~4 m/z)	0

Supplemental Figure 2. Agilent 6550 source and data-dependent acquisition settings for top-down proteomics using reverse-phase liquid chromatography and electron capture dissociation to analyze intact proteins from acetone-precipitated human brain extracts.

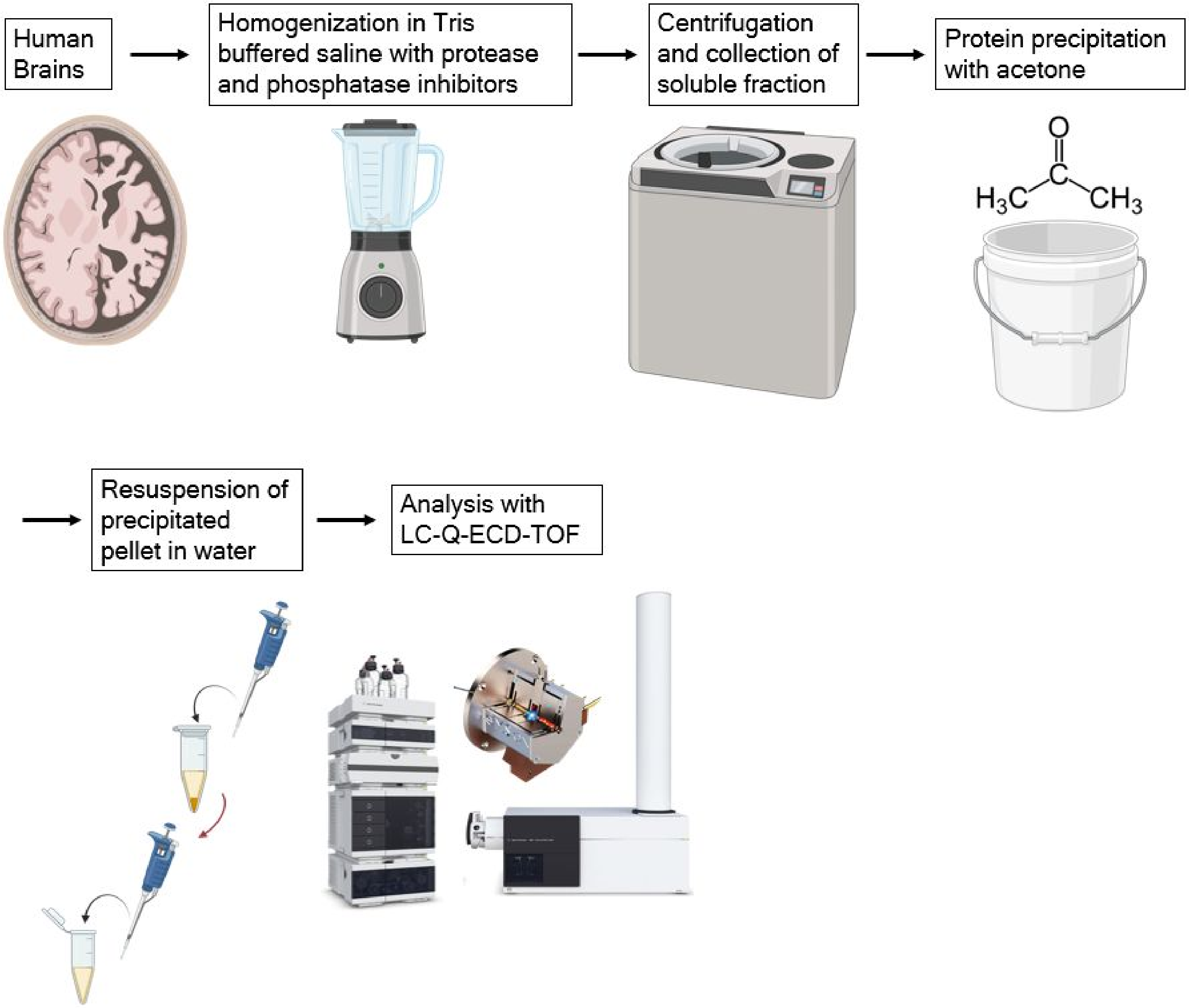

Supplemental Figure 3. Sample processing for generating a semi-complex intact protein extract from human brain tissues - sampled from healthy, Parkinson's disease, and Alzheimer's diseases cases – through homogenization, collecting soluble fraction, acetone precipitation, and resuspension of pellet in water. Figure was made using BioRender.
